# Inspired by Sea Urchins: Warburg Effect Mediated Selectivity of Novel Synthetic Non-Glycoside 1,4-Naphthoquinone-6S-Glucose Conjugates in Prostate Cancer

**DOI:** 10.3390/md18050251

**Published:** 2020-05-11

**Authors:** Sergey A. Dyshlovoy, Dmitry N. Pelageev, Jessica Hauschild, Yurii E. Sabutskii, Ekaterina A. Khmelevskaya, Christoph Krisp, Moritz Kaune, Simone Venz, Ksenia L. Borisova, Tobias Busenbender, Vladimir A. Denisenko, Hartmut Schlüter, Carsten Bokemeyer, Markus Graefen, Sergey G. Polonik, Victor Ph. Anufriev, Gunhild von Amsberg

**Affiliations:** 1 Department of Oncology, Hematology and Bone Marrow Transplantation with Section Pneumology, Hubertus Wald-Tumorzentrum, University Medical Center Hamburg-Eppendorf, 20251 Hamburg, Germany; j.hauschild@uke.de (J.H.); moritz.kaune@stud.uke.uni-hamburg.de (M.K.); tobias.busenbender@gmx.de (T.B.); c.bokemeyer@uke.de (C.B.); g.von-amsberg@uke.de (G.v.A.); 2 G.B. Elyakov Pacific Institute of Bioorganic Chemistry, Far-East Branch, Russian Academy of Sciences, 690022 Vladivostok, Russia; pelageev@mail.ru (D.N.P.); alixar2006@gmail.com (Y.E.S.); khea-96@mail.ru (E.A.K.); borisovaksenia@mail.ru (K.L.B.); vladenis@piboc.dvo.ru (V.A.D.); sergpol007@mail.ru (S.G.P.); anufriev@piboc.dvo.ru (V.P.A.); 3 School of Natural Sciences, Far Eastern Federal University, 690091 Vladivostok, Russia; 4 Martini-Klinik, Prostate Cancer Center, University Hospital Hamburg-Eppendorf, 20251 Hamburg, Germany; graefen@martini-klinik.de; 5 Institute of Clinical Chemistry and Laboratory Medicine, Mass Spectrometric Proteomics, University Medical Center Hamburg-Eppendorf, 20251 Hamburg, Germany; c.krisp@uke.de (C.K.); hschluet@uke.de (H.S.); 6 Department of Medical Biochemistry and Molecular Biology, University of Greifswald, 17489 Greifswald, Germany; simone.venz@uni-greifswald.de; 7 Interfacultary Institute of Genetics and Functional Genomics, Department of Functional Genomics, University of Greifswald, 17489 Greifswald, Germany

**Keywords:** prostate cancer, thioglucoside conjugates, natural products, sea urchins, glucose uptake

## Abstract

The phenomenon of high sugar consumption by tumor cells is known as Warburg effect. It results from a high glycolysis rate, used by tumors as preferred metabolic pathway even in aerobic conditions. Targeting the Warburg effect to specifically deliver sugar conjugated cytotoxic compounds into tumor cells is a promising approach to create new selective drugs. We designed, synthesized, and analyzed a library of novel 6-S-(1,4-naphthoquinone-2-yl)-d-glucose chimera molecules (SABs)—novel sugar conjugates of 1,4-naphthoquinone analogs of the sea urchin pigments spinochromes, which have previously shown anticancer properties. A sulfur linker (thioether bond) was used to prevent potential hydrolysis by human glycoside-unspecific enzymes. The synthesized compounds exhibited a Warburg effect mediated selectivity to human prostate cancer cells (including highly drug-resistant cell lines). Mitochondria were identified as a primary cellular target of SABs. The mechanism of action included mitochondria membrane permeabilization, followed by ROS upregulation and release of cytotoxic mitochondrial proteins (AIF and cytochrome C) to the cytoplasm, which led to the consequent caspase-9 and -3 activation, PARP cleavage, and apoptosis-like cell death. These results enable us to further clinically develop these compounds for effective Warburg effect targeting.

## 1. Introduction

Chemotherapy remains an important treatment component for the vast majority of cancer patients. However, a lack of selectivity for tumor cells over normal cells frequently results in insufficient drug concentrations in the malignant tissue, systemic toxicity, and the development of drug resistance. Therefore, inexorable efforts have been made to develop targeted therapies allowing the release of cytotoxic activity in the region of interest while sparing healthy tissue. Potential therapeutic targets include cell surface antigens, growth receptors, key regulators of cellular signal transduction, and DNA repair defects [1]. Antibody drug conjugates (ADCs) consisting of an antibody linked to a biological active payload are under intensive investigation and first drugs are available in clinics, e.g., gemtuzumab ozogamicin in acute myeloid leukemia or brentuximab vedotin in relapsed Hodgkin´s lymphoma [2]. In urothelial carcinoma, nectin-4-targeted enfortumab vedotin has been recently accepted for priority review by the FDA [3]. On a similar note, radioisotope conjugated targeting antibodies are developed for imaging and radioimmunotherapy strategies. In fact, PSMA-ligand therapy with PSMA-linked α- or β-emitters are evaluated in clinical trials in prostate cancer and are applied to patients in every day′s routine on individual bases [4]. Additional targeted therapies in prostate cancer include androgen-receptor (AR) targeting drugs such as abiraterone acetate, apalutamide, or enzalutamide as well as PARP-inhibitors in patients with DNA-repair defects [5].

Targeting the Warburg effect is an interesting novel treatment approach utilizing an increased sugar uptake of cancer cells to incorporate sugar conjugated cytotoxic compounds into tumor cells [6,7]. The high sugar demand of tumor cells results from the predominantly used metabolic pathway of glycolysis—even in aerobic conditions—which is energetically less efficient than oxidative phosphorylation found in healthy cells [8]. Thus, an overexpression of glycolytic enzymes in cytoplasm and glucose transporters (GLUTs) on the surface of cancer cells is frequently found in tumor cells in order to comply with the high sugar consumption [6,7]. 

Consequently, new compounds targeting the Warburg effect have been developed. In fact, sugar conjugates of docetaxel, busulfan, paclitaxel, and chlorambucil were found to be more selective to cancer cells in comparison with the non-conjugated “mother” molecules (reviewed in [6]). Furthermore, a glycosylated derivative of the alkylating agent ifosforamide—glufosfamide—has successfully passed clinical trials in human pancreatic, brain and CNS, lung cancer and soft tissue sarcoma [9].

More than 50% of the drugs currently available on the market are based on natural compounds and their synthetic derivatives [10]. Compared to compounds isolated from terrestrial sources, the marine-derived molecules are less studied [11,12]. At the same time, to date there are ten drugs approved for clinical use, seven of which are approved for the treatment of cancer and cancer-related conditions [13,14]. Spinochromes are an important family of polyhydroxynaphthoquinone secondary metabolites which were found in various sea urchin species. Due to their chemical diversity and promising pharmacological properties, these compounds attract the attention of scientific community as a base for development of new drugs [15,16]. Thus, spinochromes have revealed anti-allergic, anti-hypertensive, anti-diabetic, anti-oxidant, anti-inflammatory, and cardioprotective properties (reviewed in [15,16]). In general, biological effects of naphthoquinones are associated with generation of reactive oxygen species and modulation of redox signaling radical reactions [17] being either pro- or antioxidants or electrophiles [18]. Additionally, naphthazarins (5,8-dihydroxy-1,4-naphthoquinone derivatives), as well as other polymethoxylated natural and semisynthetic compounds possessing aromatic core, were reported to be potent anti-mitotic agents. Some of these compounds are capable of microtubule destabilizing and are therefore highly cytotoxic to cancer cells, representing an attractive starting point for further design as anticancer agents [19,20]. Thus, trimethyl ether of spinochrome D (tricrozarin B) inhibits a colony formation of human cancer HeLa S3 cells having IC_50_ of 0.007 μg/mL [21]. Furthermore, our previous study also showed that methoxy derivatives of 1,4-naphthoquinones exhibit greater cytotoxicity compared to the corresponding unsubstituted hydroxy analogs [22].

In this study, in continuation of our research on sea urchin pigments [23,24,25], we synthesized and analyzed novel sugar conjugates of naturally derived and chemically modified hydroxyl-1,4-naphthoquinones related to the sea urchin spinochrome pigments. Recently, we identified several natural 1,4-naphthoquinones to be active in human drug-resistant prostate cancer cells [26] and were able to synthesize first conjugates with d-glucose possessing *in vitro* cytotoxic activity [22,26]. In the current study, we further modified the compounds bearing hydroxy-1,4-naphthoquinone scaffold and investigated their anticancer properties and the mechanism of action. Thus, to increase the selectivity of the identified natural 1,4-naphthoquinones via Warburg effect targeting, we conjugated these bioactive moieties with 6-mercaptoglucose. A glycoside bond is chemically reactive and may be easy degraded in the living system via enzyme-catalyzed hydrolysis. At the same time, thioglycosides have been reported to be more resistant to the enzyme-mediated degradation [27]. Therefore, we designed and synthesized a library of non-glycoside conjugates in order to increase stability of the target compounds under human body conditions; additionally, we introduced a novel sulfur linker (thioether bond) to prevent potential hydrolysis by the human glycoside-unspecific enzymes. It is important to note that an unsubstituted glycoside hydroxy group (at C1 position) is relevant for the stabilizing of the hydrogen bond interaction between glucose and GLUT-1 and therefore for successful uptake of the glucose conjugate via this system. In contrast, the conjugation of glucose at C6 position should have a minimal impact on the GLUT-1 mediated glucose uptake and therefore on the uptake of the synthesized compounds by the cancer cells. We were able to synthesize the new acetylated (protected) and non-acetylated (unprotected, containing free-glucose scaffold) thio-conjugates of 1,4-naphthoquinone and glucose. Human drug-resistant prostate cancer cells were chosen as the main model because of the known overexpression of GLUT-1. Here, we describe the synthesis of these new conjugates, as well as their Warburg effect-guided selective anticancer activity and mode of action.

## 2. Results

### 2.1. Design and Synthesis of the 6-S-(1,4-Naphthoquinon-2-yl)-d-Glucose Chimera Molecules

In continuation of the research on synthesis of bioactive 1,4-naphthoquinones, capable of selective activity towards human drug-resistance prostate cancer cells, we designed the chimera molecules consisting of cytotoxic 1,4-naphthoquinone pharmacophore and 6-thioglucose moiety. These derivatives are expected to exhibit selective cytotoxicity to cancer cells due to Warburg effect targeting and to be more stable in human body in comparison with conventional 1,4-naphthoquinone-glucosides due to the non-glycoside bond and thioether nature of the linker.

**Figure 1:**
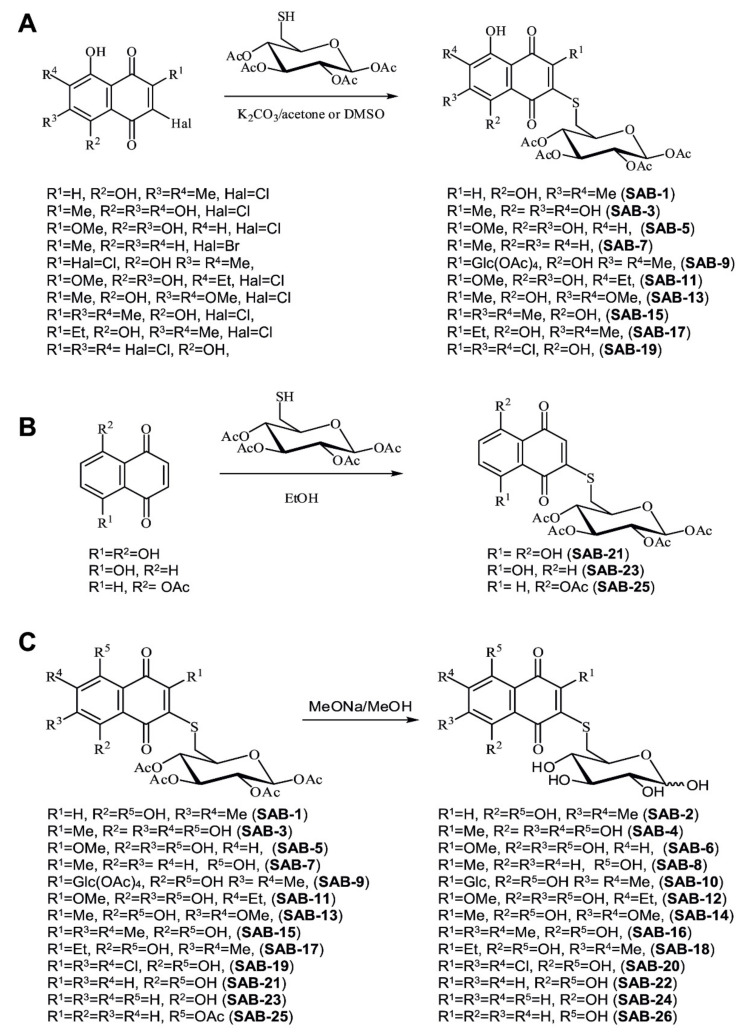
Scheme of synthesis: (**A**) synthesis of acetylated conjugates **SAB-1, -3, -5, -7, -9, -11, -13, -15, -17,** and -**19** from halogenoquinones with tetra-*O*-acetyl-6-mercaptoglucose; (**B**) synthesis of the acetylated conjugates **SAB-21, -23,** and -**25** from juglone derivatives and naphthazarin; and (**C**), synthesis of the deacetylated (unprotected) conjugates **SAB-2, -4, -6, -8, -10, -12, -14, -16, -18, -20, -22, -24,** and **-26** from the corresponding acetylated conjugates.

Thus, two different synthetic approaches were used for conjugation of naphthoquinones with 6-mercaptoglucose. We applied either: (a) a substitution reaction of halogenoquinones with readily available tetra-*O*-acetyl-6-mercaptoglucose in a basic condition (Figure 1A); or (b) an addition reaction of tetra-*O*-acetyl-6-mercaptoglucose to juglone, its acetate, or naphthazarin (Figure 1B). This was followed by saponification of the synthesized acetylated conjugates with MeONa/MeOH (Figure 1C).

Thus, halogenoquinones (Figure 1A) could be readily condensed with tetra-*O*-acetyl-6-mercaptoglucose in acetone solution with K_2_CO_3_, which resulted in acetylated conjugates **SAB-1, -3, -5, -7, -9, -11, -13, -15, -17,** and -**19** with the yields of about 60–70%. Note that chloroquinones, having an acidic β-hydroxyl group in the quinone core, did not react with tetra-*O*-acetyl-6-mercaptoglucose under the same conditions; therefore these reactions were performed in DMSO solution (yields ~80%).

As shown previously, reaction of thiols with juglone in EtOH gives exclusively 3-substituted products [28]. However, we observed that 5-acetyljuglone reacts with thiols giving the 2-substituted isomer as the main product of the reaction (Figure 1B). Boiling of naphthazarin excess with mercaptoglucose (molar ration 2:1) mainly gave 2-monosubstituted product **SAB-21** (Figure 1B).

Further saponification of the synthesized acetylated conjugates by MeONa/MeOH led to the deprotected conjugates **SAB-2, -4, -6, -8, -10, -12, -14, -16, -18, -20, -22, -24,** and **-26** with the yields of 75–82% (Figure 1C). Thus, we were able to synthesize 26 compounds (14 pairs) containing either free or acetylated 6-thioglucose moiety (Figure 1A–C).

### 2.2. Evaluation of Cytotoxicity and Selectivity

**Figure 2:**
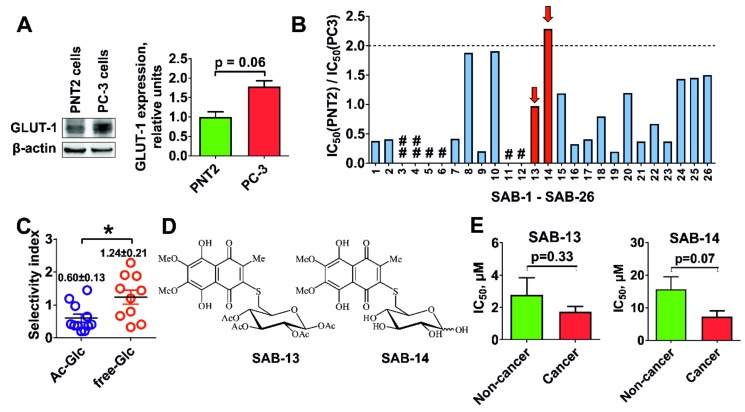
Cytotoxicity and selectivity of the synthesized compounds. (**A**) The Western blot examination of GLUT-1 expression in human prostate cancer PC-3 versus human prostate non-cancer PNT2 cells and its quantification. β-actin was used as a loading control (mean ± SEM; *n* = 3; Student′s *t*-test). (**B**) Ratio of IC_50_ (PNT2)/IC_50_ (PC-3) indicated selective cytotoxicity towards human prostate cancer cells versus human prostate non-cancer cells. “#”, IC_50_ towards one of the tested cell line was >100 µM; “##”, IC_50_s (inhibition concentrations 50%) towards both tested cell line were >100 µM. (**C**) Pooled selectivity index (SI) value of acetylated derivatives (Ac-Glc) vs. non-acetylated derivatives (free-Glc) (mean ± SEM; *n* ≥ 10; * *p* < 0.05, Student′s *t*-test). For each compound, the SI was calculated as IC_50_ (PNT2)/IC_50_ (PC-3). (**D**) Structures of compounds **SAB-13** and **-14**. (**E**) The mean cytotoxicity of the most promising compounds **SAB-13** and **-14** in five prostate cancer (LNCaP, 22Rv1, VCaP, PC-3, and DU145) vs. five non-cancer (PNT2, RWPE-1, HEK 293T, MRC-9, and HUVEC) cell lines (mean ± SEM; *n* = 5; Student′s *t*-test). Cells were treated for 48 h. The viability was measured by MTT assay.

First, we evaluated cytotoxic activity of the synthesized compounds in human drug-resistant prostate cancer PC-3 cells and human prostate non-cancer PNT2 cells. It has been reported that GLUT-1 is a main and the most abundant expressed receptor responsible for the glucose uptake in mammalian cells [7]. The expression of GLUT-1 in PC-3 was found to be higher in comparison with PNT2 cells (Figure 2A), thus these lines were considered a suitable model for the screening of Warburg-effect targeting substances (Figure 2B). Indeed, the selectivity of the conjugates bearing unprotected glucose residue (and therefore exhibiting increased affinity to GLUT-1) was significantly higher in comparison with acetylated derivatives (Figure 2C). Thus, we were able to identify one conjugate, **SAB-14**, to have a selectivity index (SI) > 2 (Figure 2B). For the further investigations, we selected **SAB-14** and **-13**—the acetylated derivative revealing a ~10-fold higher cytotoxicity and belonging to the same structural group as **SAB-14**. Both compounds were evaluated in five human prostate cancer cell lines known for different resistance profiles: LNCaP (AR-FL^+^ and AR-V7^−^, androgen-dependent, docetaxel-sensitive), 22Rv1 and VCaP (AR-FL^+^ and AR-V7^+^, androgen-independent, docetaxel-sensitive), and PC-3 and DU145 (AR-FL^−^ and AR-V7^−^, androgen-independent, docetaxel-resistant) [29,30]. In addition, five human non-cancer lines (PNT2, RWPE-1, HEK 293T, MRC-9, and HUVEC) were exposed to the derivates. In line with our previous results, both compounds were active and selective to human prostate cancer cells in comparison with non-cancer lines (Figure 2E). Note that **SAB-14**, bearing unprotected glucose moiety (Figure 2D), exhibited higher selectivity to the cancer cells (Figure 2E).

### 2.3. Cytotoxicity Correlates with Glucose Uptake Rate in Prostate Cancer Cells

**Figure 3:**
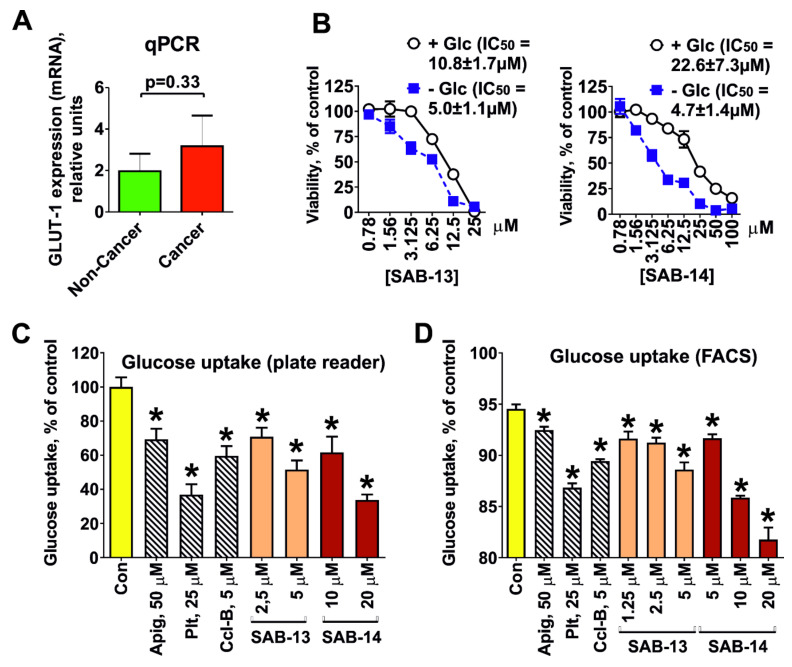
Cytotoxicity of the compounds is related to the Warburg effect. (**A**) The expression of the glucose transporter 1 mRNA (GLUT-1) in five human prostate cancer cell lines (LNCaP, 22Rv1, VCaP, PC-3, and DU145) versus four human non-cancer cell lines (PNT2, RWPE-1, HEK 293T, MRC-9, and HUVEC) (mean ± SEM; *n* = 5; Student′s *t*-test). The expression was measured by qPCR. (**B**) The viability of PC-3 cells incubated with **SAB-13** and **-14** in glucose-free (−Glc) or in glucose-containing media (+Glc, 2 g/L). Cytotoxic activity was measured using MTT test following 24 h of treatment. (**C**,**D**) Concurrent inhibition of glucose uptake by the **SAB-13** and **-14**. PC-3 cells were treated with the compounds for 24 h and then the glucose uptake was measured using 2-NBDG-based assay either in cell culture by plate reader (**C**) or in single cells using flow cytometry technique (**D**) and then normalized to cell viability (mean ± SEM; *n* = 3; * *p* < 0.05, one-way ANOVA test). Apigenin (Apig), phloretin (Plt), and cytochalasin B (Ccl-B) were used as positive controls. The viability was measured by MTS assay (**B**,**C**) or by flow cytometry using PI staining (**D**).

To examine whether synthesized compounds are able to target the Warburg effect, we accessed the glucose transporter-1 (GLUT-1) expression in the cells. Note that a mean GLUT-1 mRNA expression determined by qPCR was 1.6-fold higher in prostate cancer cells (LNCaP, 22Rv1, VCaP, PC-3, and DU145 cell lines) in comparison with human non-cancer cells (PNT2, RWPE-1, HEK 293T, MRC-9, and HUVEC cell lines), which correlated with the selectivity of the compounds (Figure 3A). Additionally, glucose depletion from culture medium resulted in increase of cytotoxic activity of both compounds (Figure 3B). Moreover, both compounds were able to inhibit glucose uptake in PC-3 cells, which was observed using two different detection methods (Figure 3C,D). Taken together, our results suggest that the novel compounds are concurrently ingested by the cells via the same GLUT-1 system as glucose and thus target the Warburg effect.

### 2.4. SAB-13 and -14 Induce Caspase-Dependent Apoptosis

**Figure 4:**
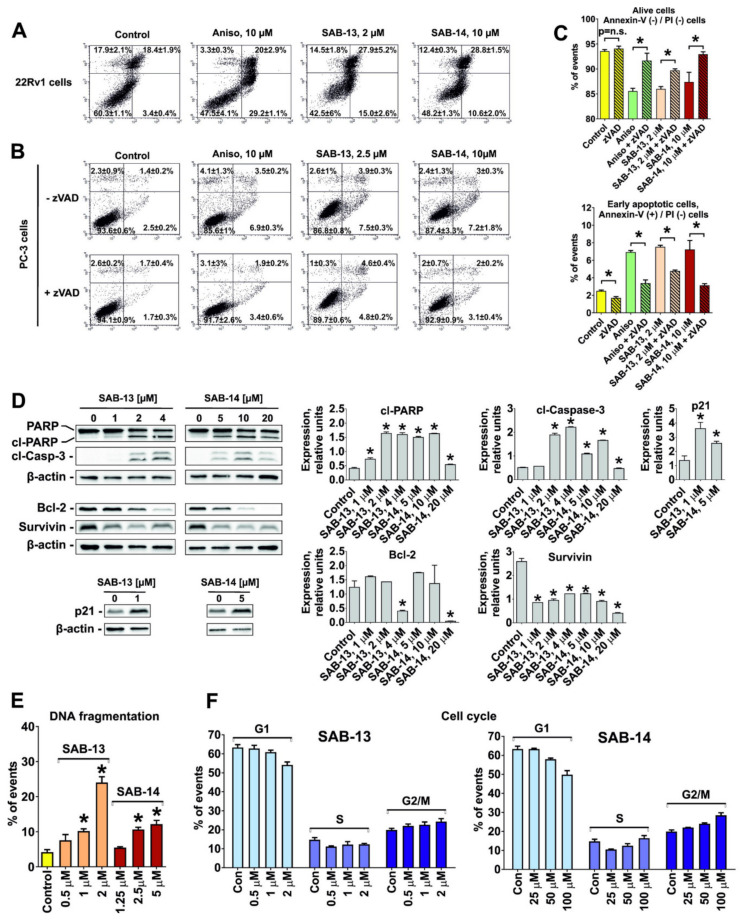
Pro-apoptotic activity of SAB-13 and SAB-14. (**A**–**C**,**E**,**F**), FACS analysis of the cells after 48 h treatment. Analysis of apoptosis induction in 22Rv1 (**A**) and PC-3 cells (**B**) using Annexin-V-FITC/propidium iodide (PI) double staining. PC-3 cells were pre-treated with 100 µM of pan-caspase inhibitor z-VAD(OMe)-fmk (zVAD) for 1 h and then treated with indicated concentrations of the drugs for 48 h (**B**). Viable cells (Annexin-V-FITC(–)/PI(–), LL quadrant) or early apoptotic cells (Annexin-V-FITC(+)/PI(–), LR quadrant) were quantified using the Cell Quest Pro software (**C**) (mean ± SEM; *n* = 3; * *p* < 0.05, Student′s *t*-test). (**D**) Western blotting analysis of the protein expression in 22Rv1 cells after 48 h of treatment. β-actin was used as a loading control (mean ± SEM; *n* = 3; one-way ANOVA test). Anisomycin (Aniso; treatment with 10 µM for 48 h) was used as a positive control. (**E**,**F**) Cell cycle analysis of 22Rv1 cells using PI staining, apoptotic cells were detected as sub-G1 population (**E**) (mean ± SEM; *n* = 3; * *p* < 0.05, one-way ANOVA test).

Pro-apoptotic signs such as phosphatidylserine externalization (Figure 4A–C) and PARP cleavage (Figure 4D) were found in the cells following 48 h treatment with **SAB-13** and **-14** at the concentrations which were close to IC_50_s in the correspondent cell lines. In addition, cleavage of caspase-3 was observed (Figure 4D). Co-treatment with pan-caspase inhibitor zVAD antagonized the cytotoxic effects of **SAB-13** and **-14**, suggesting a caspase-dependent character of the induced apoptosis (Figure 4B). Furthermore, the cell cycle analysis revealed DNA fragmentation, another pro-apoptotic marker, detected as sub-G1 peak (Figure 4E). More detailed analysis of the generated data revealed a G2/M-cell cycle arrest under drug treatment (Figure 4F). This could be at least in part explained with the observed upregulation of p21 (Figure 4D), which may contribute both to the cycle arrest as well as to the induced apoptotic cell death [31].

For this and further examinations, 22Rv1 cells were used as the main model. 22Rv1 cells express both AR-full length and AR-V7 [32]. The latter mediates resistance of prostate cancer to androgen receptor targeted therapies such as abiraterone or enzalutamide [33,34]. At the same time, PC-3 cells are AR-full length- and AR-V7-negative [32] and thus might be less relevant as clinical model for prostate cancer.

### 2.5. Effect of SAB-13 on the Proteome of Prostate Cancer Cells

To identify possible processes and molecular targets affected under the treatment, we investigated changes in proteome of 22Rv1 cells treated with **SAB-13**, as the most active compounds of the synthesized panel, at the concentration close to IC_50_s in the correspondent cell line. The changes in the protein expression were examined using the LC-MS/MS method in DIA (data independent acquisition) mode. In total, 2163 proteins were identified and quantified. In total, 253 proteins had 1.5 ≤ fold change ≤ 1/1.5 and were significantly regulated (*p* ≤ 0.05) (see Supplementary Materials Table S1). In fact, upregulation of 104 proteins and downregulation of 149 was detected following the treatment.

**Figure 5:**
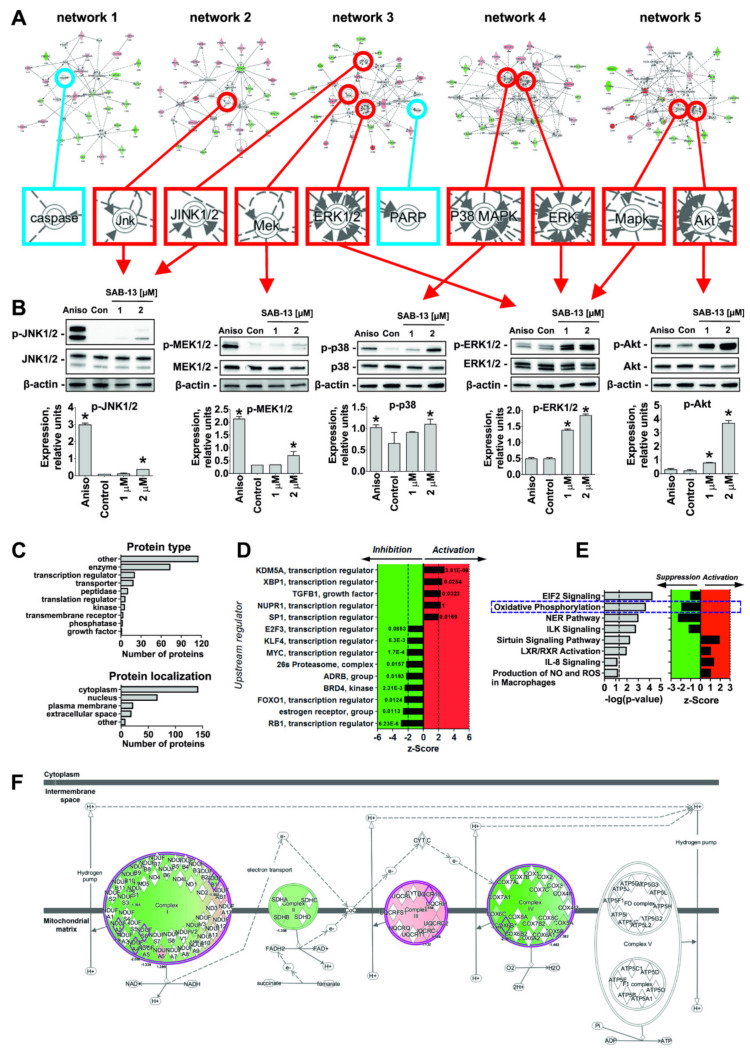
The effect of SAB-13 on proteome of prostate cancer cells. 22Rv1 cells were treated with **SAB-13** (2 µM, 48 h). The changes in proteome identified by LC-MS/MS and later analyzed using Ingenuity Pathway Analysis (IPA) software and z-score algorithm. (**A**) Hypothetical protein interaction networks between regulated proteins and the proteins predicted to be involved in interactions (constructed using IPA software). (**B**) Some kinases predicted to be affected under the treatment were further validated by Western blotting (marked by red circle). The activation was observed 2 h after the treatment. β-actin was used as a loading control. The expression phospho-kinases was normalized to the non-phospho-kinases expression levels (mean ± SEM; *n* = 3; * *p* < 0.05; one-way ANOVA test). (**C**) Gene ontology analysis. (**D**) Top upstream targets and the calculated z-score of the effect (activation/inhibition), predicted by IPA. *p*-values of overlap is indicated on the graph (significance: *p* < 0.05, Fischer′s exact test). (**E**) Top canonical pathways, predicted by IPA. The target/process is expected to be activated if z-score > 0 (red area) or suppressed if z-score < 0 (green area) (significance: *p* < 0.05, Fischer′s exact test). (**F**) Representative scheme of oxidative phosphorylation. Affected proteins discovered by proteome analysis are marked with color.

The proteomics data was analyzed using the Ingenuity Pathways Analysis software (IPA, QIAGEN Bioinformatics). Five top protein networks were constructed by IPA and the alteration of five kinases involved in several pro- and anti-apoptotic processes was predicted (Figure 5A, marked with red circle) and further validated in functional assays (Figure 5B). Thus, we showed a pronounced activation of ERK1/2, p38, and Akt kinases upon the treatment, while only slight phosphorylation of MEK1/2 and JNK1/2 kinases was observed (Figure 5B). Thus, alteration of ERK1/2, p38, and Akt may play an important role in the cellular effect mediated by the synthesized compounds. Note that the regulation (cleavage) of PARP and caspase (caspase-3), predicted in IPA analysis (Figure 5A), was confirmed experimentally as well (Figure 4D).

Next, gene ontology analysis and z-score algorithm were used to further predict the effect on biological processes/target (Figure 5C). Most of the proteins regulated under the treatment were located in cellular cytoplasm and nucleus, and were identified as enzymes (Figure 5C). Remarkably, the activity of several upstream regulators playing an important role in prostate cancer development and progression have been predicted to be affected by **SAB-13** (Figure 5D). Among them, the following are suppressed: E2F3 (transcription factor, its activity was reported to be associated with poor prostate cancer patients treatment prognosis [35]), ADRB (β-adrenergic receptor, its signaling is involved in the development of aggressive prostate cancer [36]), BRD4 (kinase, associated with tumor growth and metastatic potential of prostate cancer [37,38]), 25s proteasome (a promising and relevant target in a number of human cancers including prostate cancer [39]), and MYC (proto-oncogene, is involved in initiation and progression of a number of human cancers [40] and drives the development of the highly aggressive neuroendocrine prostate cancer [36]) (Figure 5D). Additionally, sirtuin and IL-8 signaling, as well as LXR/RXR and NO and ROS production in macrophages were proposed to be suppressed, while NER and EIF2 pathway, ILK signaling, and oxidative phosphorylation were predicted to be activated under the treatment (Figure 5E). At the same time, it is important to note that predicted activation of KDM5A and XBP1 and suppression of FOXO1 and RB1 were observed. These alterations may play a possible negative role in prostate cancer as undesirable effects of the drug, which is described elsewhere. Therefore, these effects should be carefully investigated before further development of these drugs for in-human trials.

Note that we discovered the alterations of protein components of four (out of five) main protein complexes located in the inner mitochondrial membrane and involved in the oxidative phosphorylation (see Table S1). These molecules were identified as parts of Complex I (genes *NDUFA4*, *NDUFS6*, *NDUFB1*, and *NDUFA2*), Complex II (gene *SDHB*), Complex III (gene *UQCR10*), and Complex IV (genes *COX6B1* and *COX17*). Therefore, oxidative phosphorylation was predicted to be inhibited/disrupted in **SAB-13**-treated cells (z-score = −1.89, *p*-value = 2.37 × 10^−4^; Figure 5F).

### 2.6. SAB-13 and SAB-14 Induce Apoptosis via Mitochondria Targeting

**Figure 6:**
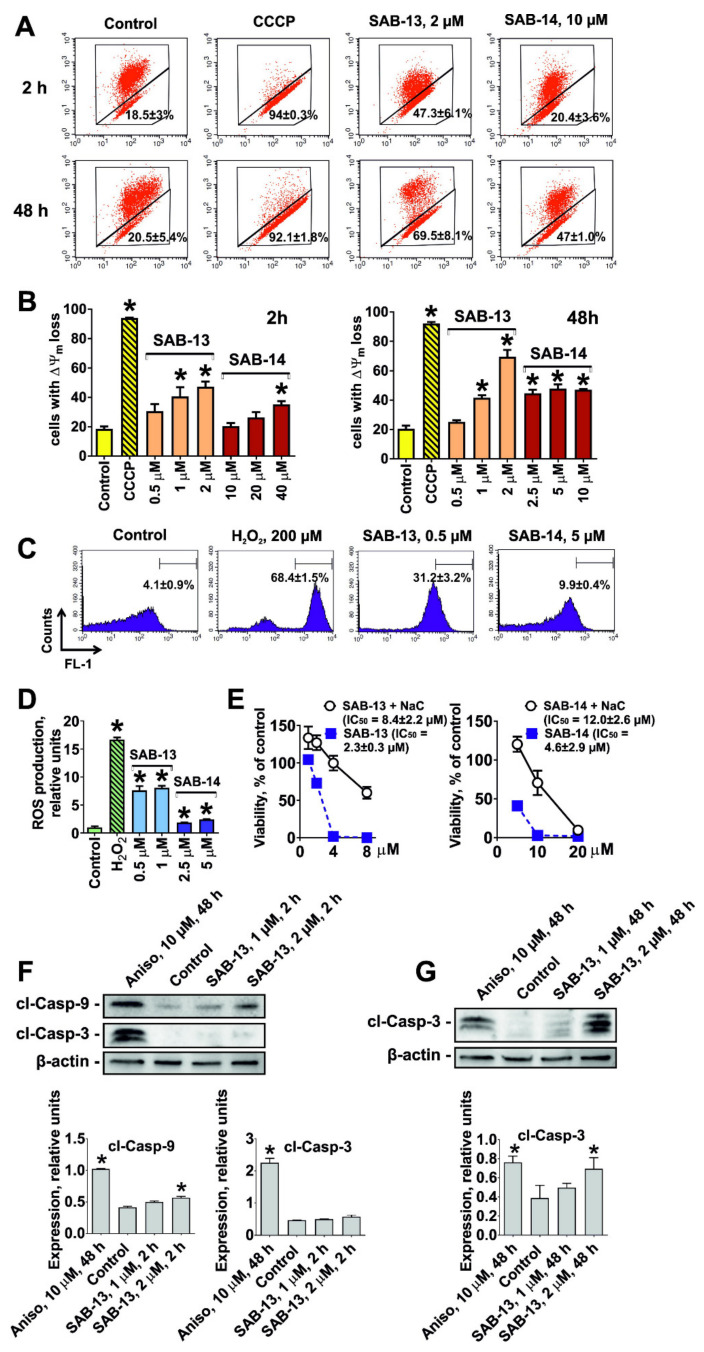
Effect of SAB-13 and SAB-14 on mitochondrial membrane potential and ROS production. (**A**,**B**) Effect of the drugs on mitochondrial membrane potential (MMP, ΔΨ_m_). 22Rv1 cells were treated with the **SAB-13** or **SAB-14** for indicated time, harvested with trypsin, stained with JC-1, and measured by FACS (**A**), and the cells containing depolarized mitochondria were quantified using the Cell Quest Pro software (**B**) (mean ± SEM; *n* = 3; * *p* < 0.05, one-way ANOVA test). CCCP (50 µM) was used as a positive control. (**C**,**D**) Effect on ROS production in 22Rv1 cells after 2 h of treatment. Cells were stained with CM-H_2_DCFDA, treated with the drugs, harvested, and analyzed by FACS (**C**), and the ROS level was quantified using the Cell Quest Pro software (**D**) (mean ± SEM; *n* = 3; * *p* < 0.05, one-way ANOVA test). H_2_O_2_ (200 µM) was used as a positive control. (**E**) 22Rv1 cells were pre-treated with 1 mM NaC (*N*-acetyl-l-cysteine) for 1 h and then co-treated with the investigated drugs for 48 h FBS- and glucose-free media. Cell viability was measured by MTT assay, treatment time was 48 h. (**F**,**G**), Western blotting analysis of cleaved caspase-3 and -9 levels in 22Rv1 cells treated with **SAB-13** for 2 h (**F**) or 48 h (**G**). Anisomycin (Aniso) was used as a positive control. β-actin was used as a loading control (mean ± SEM; *n* = 3; one-way ANOVA test).

**Figure 7:**
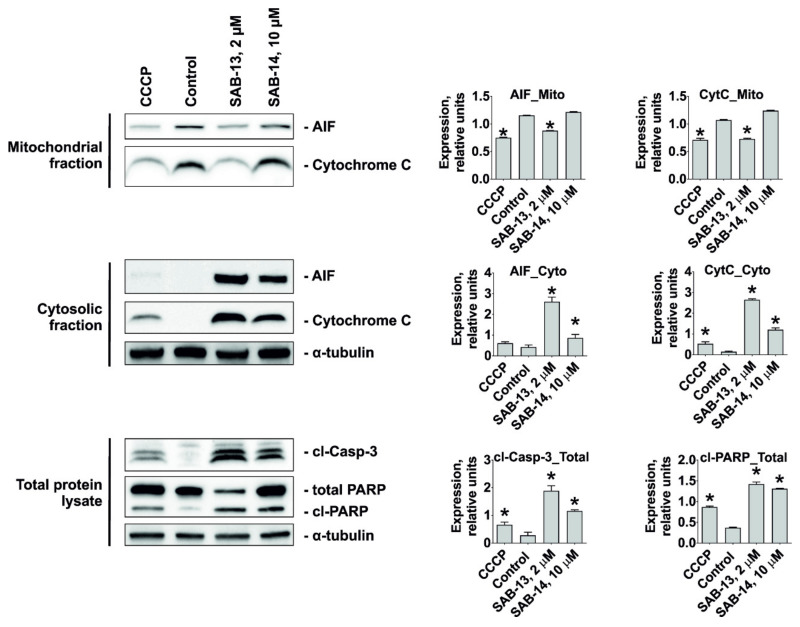
Quantification of the proteins in mitochondrial, cytoplasmic, and total fractions. 22Rv1 cells were treated with the drugs for 48 h, the proteins were extracted and fractionated using Cell Fractionation Kit (abcam). The mitochondrial (Mito), cytoplasmatic (Cyto) or total (Total) fractions were concentrated and analyzed by Western blotting. Cells treated with carbonyl cyanide 3-chlorophenylhydrazone (CCCP; 50 µM) were used as a positive control. α-tubulin was used as a loading control, the protein expression was normalized to α-tubulin expression levels either in cytosolic fraction or in the total protein lysate (mean ± SEM; *n* = 3; * *p* < 0.05; one-way ANOVA test).

Oxidative phosphorylation (or electron transport-linked phosphorylation) is a metabolic pathway of nutrients oxidation which takes place in mitochondria and ultimately results in ATP production in eukaryotic cells [41]. Thus, its disruption predicted by bioinformatical analysis of proteomics data may indicate mitochondria targeting by **SAB-13** and similar compounds. **SAB-13** was found to affect complexes I-IV, essential elements of oxidative phosphorylation machinery, which are located in the mitochondrial inner membrane. Consequently, we examined the effect of **SAB-13** on the mitochondrial membrane potential (ΔΨ_m_) at the concentration close to IC_50_. Significant and rapid loss of ΔΨ_m_ was detected in the prostate cancer cells following both short- (2 h) and long-term (48 h) treatment (Figure 6A,B). Next, ROS production level was examined, which is known to be closely associated with mitochondrial integrity [42]. Indeed, a significant increase of the ROS level was observed 2 h after the treatment, simultaneously with ΔΨ_m_ loss (Figure 6C,D). Moreover, pretreatment with an established antioxidant N-acetyl-L-cysteine could rescue the cells from the drug-induced apoptosis (Figure 6E). This confirms the cytotoxic effect of ROS generation under treatment. Additionally, we were able to show that caspase-9 (but not caspase-3) is activated shortly after treatment at the concentration close to IC_50_ (2 h, Figure 6F), whereas caspase-3 cleavage was observed after 48 h treatment (Figure 6G). Finally, the examination of different subcellular fractions revealed a redistribution of such pro-apoptotic proteins as cytochrome C and AIF (apoptosis inducing factor) from mitochondria to cytoplasm (Figure 7). This effect was accompanied by apoptosis induction, as indicated by cleaved PARP and cleaved caspase-3 as apoptotic markers (Figure 7). In conclusion, induced cancer cell apoptosis was strongly associated with targeting of mitochondria.

## 3. Discussion

A significant percentage of prostate cancer patients initially respond to targeted (e.g., abiraterone acetate, enzalutamide, PARP-inhibitors, and PSMA-ligand therapy) or untargeted therapies (e.g., cabazitaxel, docetaxel, and radium-223) [43]. However, the majority of patients will experience an increasing loss of sensitivity to standard medications with each additional treatment line [44]. Primary as well as secondary drug-resistance are responsible for the limited treatment success and poor prognosis of patients suffering from advanced castration resistant prostate cancer (CRPC) [45]. Thus, expression of androgen receptor transcriptional variants of AR (AR-Vs), in particular AR-V7 [33], has been identified as an important mechanism of resistance to AR-targeted therapies. In addition, side effects caused by the damage of non-malignant cells limit the application of standard therapies in a mostly older and comorbid patient population. Thus, novel therapeutics capable of overcoming drug resistance while sparing side effects are urgently needed.

Conjugation of the cytotoxic molecule and the moiety, which is responsible for selective delivery of the active substance to cancer cells may increase specificity of the drug and to reduce its side effects. In the current study we conjugated bioactive 1,4-naphthoquinone scaffold, which has been previously shown to exhibit cytotoxic activity in human cancer cell lines, with a glucose moiety via a sulfur linker at C6 position using simple and effective methods. Note that the generated non-glycoside bond simplified the chemical synthesis of the compounds. Thus, we recently reported a number of O-glycosides of 1,4-naphthoquinones to undergo decomposition during the deacetylation step [46]. Moreover, we showed that some S-glycosides can convert into linear or angular tetracycles under the same conditions [46], whereas no undesired effects or side reactions were observed during the synthesis described in the current research. Thus, we used either: (i) a substitution reaction of the halogen atoms in 1,4-naphthoquinone derivatives by a sulfur atom of 6-mercaptoglucose derivative; or (ii) an addition reaction of 6-mercaptoglucose derivative to juglone, its acetate, or naphthazarin, followed by saponification of the acetylated conjugates. 

This modification resulted in a higher specificity to cancer cells due to Warburg effect targeting as well as increased water solubility and therefore bioavailability of the synthesized molecules. An exchange of the ether linker to the thioether linker most likely will provide higher stability of the compounds in the human body [27] and a greater resistance to the enzymes mediated hydrolysis. The conjugation at C6 position of glucose molecule ensures the affinity of the synthesized conjugates to GLUT-1 and therefore its successful cellular uptake via this system. Furthermore, it should provide additional resistance to glycoside-specific enzymes due to the non-glycoside nature of the bond as well as novel sulfur linker.

Thus, we could synthesize 14 acetylated derivatives and 14 corresponding water-soluble unprotected (deacetylated) conjugates. Following a screening of the synthesized compounds, we chose two most promising candidates. Hence, **SAB-14** (6-((6-deoxy-6-S-d-glucopyranosyl)thio)-5,8-dihydroxy-7-methyl-2,3-dimethoxy-1,4-naphthoquinone) and its acetylated analog **SAB-13** (6-((1,2,3,4-tetra-*O*-acetyl-6-deoxy-*β*-d-glucopyranosyl)thio)-5,8-dihydroxy-7-methyl-2,3-dimethoxy-1,4-naphthoquinone) exhibited selectivity to human prostate cancer cells and were chosen for further investigations. The selectivity of these drugs was mediated by Warburg effect. This was confirmed by several experiments, namely correlation of selectivity to cancer cell lines with increased GLUT-1 expression in these cells, the increased cytotoxicity of the compounds in the glucose-depleted media, and the ability of the compounds to inhibit glucose uptake by the cells, which was confirmed with two different methods. Additionally, the compounds bearing unsubstituted glucose residue (e.g., **SAB-14**) were generally more selective in comparison with its acetylated analogs (e.g., **SAB-13**). This can be explained by the higher affinity of unprotected glucose residue to GLUT-1. These results strongly suggest the synthesized drugs were taken up by the same system, as glucose, i.e., glucose transporters (GLUTs).

**Figure 8:**
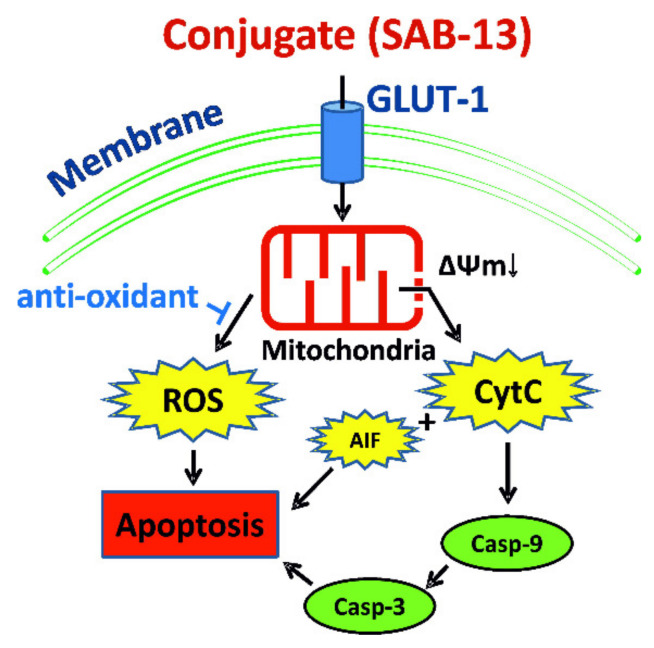
Suggested mechanism of anticancer activity *in vitro*.

The mechanism of anticancer activity of the natural 1,4-naphthoquinones and other related compounds includes generation of free superoxide radicals, which results in DNA damage and may lead to p53-independent cell death [22,47,48], as well as inhibition of topoisomerase-II [47,49,50]. Consequently, the examination of the mechanisms of anticancer activity of **SAB-13** and **-14** revealed an induction of caspase-dependent apoptosis. Additionally, we detected a G2/M arrest of the cancer cells under drug treatment, which could be related to the observed p21 upregulation in the treated cells. Pro-apoptotic effects of the synthesized conjugates were accompanied by downregulation of several anti-apoptotic proteins. Further investigation of the drug-mediated changes in cellular proteome as well as bioinformatical analysis of the data suggested mitochondria targeting as one of the central effects of **SAB-13**. Indeed, we were able to validate this effect in functional assays. Thus, **SAB-13** is able to promote a generation of cytotoxic ROS and to induce a drop-down of mitochondrial membrane potential, which resulted in a release of cytotoxic mitochondrial proteins to cellular cytoplasm, caspases activation and ultimately cancer cell death (Figure 8). In addition, **SAB-13** activated caspase-9 shortly after treatment and prior to caspase-3 (Figure 8). It has been previously shown that the modification of existing drugs in order to assign them mitochondria-targeting properties (i.e., conjugation with the mitochondria-specific carrier molecule) significantly increases their activity towards cancer cells [51,52]. Moreover, these drugs were reported to be able to overcome the drug resistance to standard medications [51,52]. Despite the upregulated glycolysis (anaerobic respiration) in cancer cells in comparison to normal tissues, the oxidative phosphorylation (part of aerobic respiration machinery) was reported to be still active and even upregulated in different cancer entities [53]. Thus, oxidative phosphorylation has recently been identified as a new promising target in anticancer therapy promoting the therapeutic potential of the inhibitors of this biochemical process [41]. In fact, inhibitors of different steps of the oxidative phosphorylation cascade such as metformin, atovaquone, arsenic trioxide, carboxyamidotriazole, fenofibrate, and lonidamine have exhibited therapeutic efficacy in a number of clinical and preclinical studies for the treatment of different cancer types (reviewed in [53]). Thus, the combination of Warburg effect mediated selectivity and the simultaneous targeting of oxidative phosphorylation/mitochondria in cancer cells makes the synthesized conjugates promising agents in the therapy of the human CRPC and other cancers. Ongoing experiments will clarify if oxidative phosphorylation is a primary target of the drugs, or if the targeting of other mitochondrial components and functions cause a disruption of the oxidative phosphorylation along with other mitochondrial processes. In vivo experiments are currently in preparation to validate the above-described *in vitro* results.

## 4. Materials and Methods

### 4.1. Chemistry

#### 4.1.1. General Chemistry (Reagents, Solvents and Equipment)

Reagents and solvents were purchased from Sigma (Taufkirchen, Germany) and Vekton (St. Petersburg, Russia). The initial quinones were purchased from Sigma or synthesized as described elsewhere. All reagents, initial quinones, and solvents were of analytical grade and used as received. The melting points were determined on a Boetius melting-point apparatus (Dresden, Germany) and are uncorrected. The ^1^H and ^13^C NMR spectra were recorded using Bruker Avance-300 (300 MHz), Bruker Avance III-500 HD (500 MHz), and Bruker Avance III-700 (700 MHz) spectrometers (Bruker Corporation, Bremen, Germany) using CDCl_3_ and DMSO-d_6_ as the solvents with the signal of the residual non-deuterated solvent as the internal reference. The progress of reaction was monitored by thin-layer chromatography (TLC) on Sorbfil plates (IMID, Krasnodar, Russia) using the following solvent systems as eluents: hexane/benzene/acetone, 3:1:1 (v/v) (System A) and benzene/ethyl acetate/methanol, 7:4:2 (System B). Individual substances were isolated and purified using crystallization, column chromatography as well as preparative TLC on silica gel (Silicagel 60, 0.040–0.063 mm, Alfa Aesar, Karlsruhe, Germany) which was preliminarily treated with hydrochloric acid (pH = 2) in order to reduce a residual adsorption of quinones, followed by drying and activation by heating at 120 °C.

The initial quinone *6-chloro-5,8-dihydroxy-2,3-dimethoxy-7-methyl-1,4-naphthoquinone* was synthesized from the commercially available reagents as described below. A solution of diazomethane in Et_2_O (0.2 M) was added dropwise to a stirring solution of 6-chloro-2,3,5,8-tetrahydroxy-7-methyl-1,4-naphthoquinone (299 mg, 1.0 mmol) in 1,4-dioxane (50 mL), until TLC indicated completion of dimethoxylated product with *R_f_* = 0.70 (system A) formation. The reaction mixture was evaporated in vacuo and the solid was recrystallized from methanol. Red-brown solid; yield 75% (224 mg); mp 179-181 °C; *R_f_* = 0.70 (A). ^1^H NMR (CDCl_3_, 500 MHz) *δ*: 2.42 (s, 3H, -CH_3_), 4.12 (s, 3H, -OCH_3_), 4.13 (s, 3H, -OCH_3_), 12.94 (s, 1H, C(8)-OH), 13.04 (s, 1H, C(5)-OH). ^13^C NMR (125 MHz, CDCl_3_) δ: 13.5 (-CH_3_), 61.7 (2 × -OCH_3_), 108.0 (C-8a), 108.4 (C-4a), 134.2 (C-6), 139.0 (C-7), 147.6, 148.0, 156.3 (C-5), 159.2 (C-8), 181.8 (C-4), 181.9 (C-1); IR (CHCl_3_) *ν*_max_: 3003, 2954, 2855, 1605, 1457, 1432, 1418, 1401, 1379, 1286, 1188, 1158, 1138, 1113, 1053 cm^−1^. HRMS (EI): *m*/*z* [M − H]^−^ calcd for C_13_H_10_ClO_6_: 297.0171; found: 297.0168.

*1,2,3,4-Tetra-O-acetyl-6-deoxy-6-thio-β-D-glucopyranose*, used for the synthesis of the conjugates, was synthesized from the commercially available reagents as described below. Potassium metabisulfite (4.37 g, 19.65 mmol, 1.5 eq) was added to a refluxing solution of 1,2,3,4-tetra-*O*-acetyl-6-deoxy-β-d-glucopyranosyl-6-isothiouronium iodide (7.0 g, 13.1 mmol) in mixture of water (75 mL) and chloroform (75 mL). The reaction mixture was refluxed for 2 h, cooled to room temperature. The organic layer was separated. The aqueous layer was extracted with chloroform (2 × 50 mL). The organic layers were combined, dried over NaSO_4_, filtered, and the solvent evaporated under vacuum. The residue was recrystallized from MeOH to give pure crystalline product. White solid; yield 57.4% (2.74 g); mp 109-111 °C; *R_f_* = 0.51 (A). ^1^H NMR (500 MHz, CDCl_3_) δ: 1.75 (dd, *J* = 9.6, 7.5 Hz, 1H, *-*SH), 2.01 (s, 3H, -COCH_3_), 2.03 (s, 3H, -COCH_3_), 2.05 (s, 3H, -COCH_3_), 2.12 (s, 3H, -COCH_3_), 2.62 (m, H-6a), 2.71 (m, H-6b), 3.96 (ddd, *J* = 9.7, 6.5, 3.0 Hz, 1H, H-5), 5.11 (t, *J* = 9.7 Hz, 1H, H-4), 5.12 (t, *J* = 9.4 Hz, 1H, H-2), 5.25 (t, *J* = 9.4 Hz, 1H, H-3), 5.71 (t, *J* = 8.4 Hz, 1H, H-1). ^13^C NMR (125 MHz, CDCl_3_) δ: 20.5 (2 × -COCH_3_), 20.6 (-COCH_3_), 20.8 (-COCH_3_), 25.8 (C-6), 70.3 (C-2), 70.4 (C-4), 72.8 (C-3), 75.0 (C-5), 91.7 (C-1), 169.0 (-COCH_3_), 169.2 (-COCH_3_), 169.5 (-COCH_3_), 170.1 (-COCH_3_). IR (CHCl_3_) *ν*_max_: 3056, 3006, 1759, 1602, 1429, 1370, 1250, 1192, 1079, 1038 cm^−1^. HRMS (ESI): *m*/*z* [M + Na]^+^ calcd for C_14_H_20_NaO_9_S: 387.0720; found: 387.0717.

#### 4.1.2. General Procedure for Synthesis of the Acetylated Conjugates **SAB-1**, **-7**, **-9**, **-13**, **-15**, **-17**, and **-19** in Acetone Solution (Figure 1A)

Finely powdered potassium carbonate (0.415 g, 3.0 mmol) was added to a solution of the correspondent quinone, (1.0 mmol) and tetra-*O*-acetyl-6-mercaptoglucose (0.368 g, 1.01 mmol) in acetone (120 mL). The mixture was stirred at room temperature until reaction completed (3 h, controlled by TLC), acidified with HCl, and concentrated in vacuo. The residue was purified by column chromatography (silica gel, hexane/acetone, 10:1) to give products **1, 7, 9, 13, 15, 17,** and **19**.

*2-(1,2,3,4-Tetra-O-acetyl-6-deoxy-β-d-glucopyranos-6-ylthio)-5,8-dihydroxy-6,7-dimethyl-1,4-naphthoquinone* (**SAB-1**). Red solid; yield 406 mg (70%); mp 203–205 °C; *R_f_* = 0.33 (A). ^1^H NMR (CDCl_3_, 500 MHz) δ: 2.02 (s, 3H, -COCH_3_), 2.03 (s, 3H, -COCH_3_), 2.08 (s, 3H, -COCH_3_), 2.13 (s, 3H, -COCH_3_), 2.22 (s, 3H, C(7)-CH_3_), 2.23 (s, 3H, C(6)-CH_3_), 3.05 (dd, *J* = 14.0, 7.0 Hz, 1H, H-6′a), 3.13 (dd, *J* = 14.0, 3.2 Hz, 1H, H-6′b), 3.97 (ddd, *J* = 9.5, 7.0, 3.2 Hz, 1H, H-5′), 5.11 (t, *J* = 9.5 Hz, 1H, H-4′), 5.14 (dd, *J* = 9.5, 8.2 Hz, 1H, H-2′), 5.28 (t, *J* = 9.5 Hz, 1H, H-3′), 5.73 (d, *J* = 8.2 Hz, 1H, H-1′), 6.75 (s, 1H, H-3), 13.01 (s, 1H, C(8)-OH), 13.02 (s, 1H, C(5)-OH). ^13^C NMR (125 MHz, CDCl_3_) δ: 12.3 (C(7)-CH_3_), 12.5 (C(6)-CH_3_), 20.5 (2 × -COCH_3_), 20.6 (-COCH_3_), 20.7 (-COCH_3_), 32.0 (C-6′), 70.1 (C-2′), 71.0 (C-4′), 72.6 (C-3′), 73.4 (C-5′), 91.5 (C-1′), 108.5 (C-4a), 109.4 (C-8a), 125.6 (C-3), 139.9 (C-7), 142.0 (C-6), 148.6 (C-2), 168.8 (-COCH_3_), 169.2 (-COCH_3_), 169.4 (C-5), 169.6 (-COCH_3_), 170.0 (-COCH_3_), 170.6 (C-8), 171.8 (C-1), 173.0 (C-4). IR (CHCl_3_) *ν*_max_: 2942, 1760, 1599, 1555, 1431, 1371 cm^−1^. HRMS (EI): *m/z* [M + Na]^+^ calcd for C_26_H_28_O_13_SNa: 603.1143; found: 603.1142.

*2-(1,2,3,4-Tetra-O-acetyl-6-deoxy-β-d-glucopyranos-6-ylthio)-5-hydroxy-3-methyl-1,4-naphthoquinone* (**SAB-7**). Yellow solid; yield 385 mg (70%); mp 160–161 °C; *R_f_* = 0.38 (A). ^1^H NMR (CDCl_3_, 500 MHz) δ: 1.62 (s, 3H, -COCH_3_), 1.97 (s, 3H, -COCH_3_), 1.98 (s, 3H, -COCH_3_), 2.05 (s, 3H, -COCH_3_), 2.36 (s, 3H, C(3)-CH_3_), 3.22 (dd, *J* = 14.8, 2.3 Hz, 1H, H-6′a), 3.54 (dd, *J* = 14.8, 8.3 Hz, 1H, H-6′b), 3.82 (ddd, *J* = 9.4, 8.3, 2.3 Hz, 1H, H-5′), 4.98 (t, *J* = 9.4 Hz, 1H, H-4′), 5.00 (dd, *J* = 9.4, 8.3 Hz, 1H, H-2′), 5.17 (t, *J* = 9.4 Hz, 1H, H-3′), 5.49 (d, *J* = 8.3 Hz, 1H, H-1′), 7.21 (dd, *J* = 7.2, 2.2, 1H, H-6), 7.56 (t, *J* = 7.2, 1H, H-7), 7.58 (dd, *J* = 7.2, 2.2, 1H, H-8), 12.15 (s, 1H, C(5)-OH). ^13^C NMR (CDCl_3_, 500 MHz) δ: 14.5 (C(3)-CH_3_), 19.9 (-COCH_3_), 20.5 (-COCH_3_), 20.5 (-COCH_3_), 20.6 (-COCH_3_), 33.8 (C-6′), 70.0 (C-2′), 70.6 (C-4′), 72.6 (C-3′), 76.4 (C-5′), 91.5 (C-1′), 114.9 (C-4a), 119.6 (C-8), 123.8 (C-6), 133.0 (C-8a), 135.7 (C-7), 146.4 (C-3), 147.2 (C-2), 161.3 (C-5), 168.6 (-COCH_3_), 169.1 (-COCH_3_), 169.4 (-COCH_3_), 170.0 (-COCH_3_), 180.2 (C-1), 187.2 (C-4).IR (CHCl_3_) *ν*_max_:3096, 2944, 1760, 1667, 1632; 1599, 1572, 1457, 1430, 1367cm^−1^. HRMS (ESI): *m*/*z* [M + Na]^+^ calcd for C_25_H_26_O_12_SNa: 573.1037; found: 573.1040.

*2,3-Di(1,2,3,4-tetra-O-acetyl-6-deoxy-β-d-glucopyranos-6-ylthio)-5,8-dihydroxy-6,7-dimethyl-1,4-naphthoquinone* (**SAB-9**). Red solid; yield 258 mg (65%, recovery of starting quinone was 166 mg (58%)); mp 202–205 °C; *R_f_* = 0.76 (A). ^1^H NMR (CDCl_3_, 700 MHz) δ: 1.81 (s, 6H, 2×-COCH_3_), 1.99 (s, 6H, 2 × -COCH_3_), 2.00 (s, 6H, 2 × -COCH_3_), 2.08 (s, 6H, 2 × -COCH_3_), 2.26 (s, 6H, C(6)-CH_3_, C(7)-CH_3_), 3.47 (dd, *J* = 14.5, 8.7 Hz, 2H, H-6′a, H-6′′a), 3.52 (dd, *J* = 14.5, 2.4 Hz, 2H, H-6′b, H-6′′b), 4.05 (ddd, *J* = 9.5, 8.7, 2.4 Hz, 2H, H-5′, H-5′′), 4.96 (t, *J* = 9.5 Hz, 2H, H-4′, H-4′′), 5.05 (dd, *J* = 9.5, 8.4 Hz, 2H, H-2′, H-2′′), 5.32 (t, *J* = 9.5 Hz, 2H, H-3′, H-3′′), 5.72 (d, *J* = 8.4 Hz, 2H, H-1′, H-1′′), 13.23 (s, 2H, C(5)-OH, C(8)-OH). ^13^C NMR (176 MHz, CDCl_3_) δ: 12.4 (C(6)-CH_3_, C(7)-CH_3_), 20.3 (2×-COCH_3_), 20.6 (2 × -COCH_3_), 20.6 (2 × -COCH_3_), 20.7 (2 × -COCH_3_), 35.4 (C-6′, C-6′′), 70.4 (C-2′, C-2′′), 71.3 (C-4′, C-4′′), 72.5 (C-3′, C-3′′), 75.6 (C-5′, C-5′′), 91.3 (C-1′, C-1′′), 109.3 (C-4a, C-8a), 138.6 (C-6, C-7), 145.5 (C-2, C-3), 161.7 (C-5, C-8), 168.8 (2 × -COCH_3_), 169.2 (2 × -COCH_3_), 169.7 (2 × -COCH_3_), 170.1 (2 × -COCH_3_), 178.7 (C-1, C-4). IR (CHCl_3_) *ν*_max_: 3053, 2946, 1759, 1600, 1487, 1427, 1370 cm^−1^. HRMS (EI): *m/z* [M + Na]^+^ calcd for C_40_H_46_O_22_S_2_Na: 965.1814;found: 965.1807.

*6-(1,2,3,4-Tetra-O-acetyl-6-deoxy-β-D-glucopyranos-6-ylthio)-5,8-dihydroxy-7-methyl-2,3-dimethoxy-1,4-naphthoquinone* (**SAB-13**). Dark red solid; yield 457 mg (73%); mp 96–98 °C; *R_f_* = 0.35 (A). ^1^H NMR (CDCl_3_, 500 MHz) δ: 1.99 (s, 6H, 2 × -COCH_3_), 2.00 (s, 3H, -COCH_3_), 2.02 (s, 3H, -COCH_3_), 2.51 (s, 3H, -CH_3_), 3.16 (dd, *J* = 14.5, 8.1 Hz, 1H, H-6′a), 3.36 (dd, *J* = 14.5, 2.5 Hz, 1H, H-6′b), 3.71 (ddd, *J* = 9.5, 8.1, 2.5 Hz, 1H, H-5′), 4.12 (s, 3H, -OCH_3_), 4.13 (s, 3H, -OCH_3_), 4.98 (t, *J* = 9.5 Hz, 1H, H-4′), 5.05 (dd, *J* = 8.3, 9.5 Hz, 1H, H-2′), 5.17 (t, *J* = 9.5 Hz, 1H, H-3′), 5.60 (t, *J* = 8.3 Hz, 1H, H-1′), 13.02 (s, 1H, C(8)-OH), 13.43 (s, 1H, C(5)-OH). ^13^C NMR (125 MHz, CDCl_3_) δ: 15.0 (-CH_3_), 20.5 (3×-COCH_3_), 20.6 (-COCH_3_), 34.5 (C-6′), 61.6 (-OCH_3_), 61.7 (-OCH_3_), 70.2 (C-2′), 70.9 (C-4′), 72.6 (C-3′), 74.9 (C-5′), 91.4 (C-1′), 107.8 (C-4a), 108.5 (C-8a), 136.7 (C-6), 144.7 (C-7), 147.8 (C-3), 147.9 (C-2), 160.0 (C-8), 161.3 (C-5), 168.6 (-COCH_3_), 169.2 (-COCH_3_), 169.5 (-COCH_3_), 170.0 (-COCH_3_), 180.7 (C-1), 181.0 (C-4). IR (CHCl_3_) *ν*_max_: 3698, 3610, 3026, 2947, 1760, 1602, 1558, 1455, 1433, 1392, 1374, 1286, 1250, 1192, 1136, 1114, 1077, 1038 cm^−1^. HRMS (ESI): *m*/*z* [M + Na]^+^ calcd for C_27_H_30_NaO_15_S: 649.1198; found: 649.1195.

*6-(1,2,3,4-Tetra-O-acetyl-6-deoxy-β-d-glucopyranos-6-ylthio)-5,8-dihydroxy-2,3,7-trimethyl-1,4-naphthoquinone***(SAB-15)**. Dark red solid; yield 421 mg (71%); mp 148–151 °C; *R_f_* = 0.52 (A). ^1^H NMR (CDCl_3_, 500 MHz) δ: 1.88 (s, 3H, -COCH_3_), 1.99 (s, 6H, 2 × -COCH_3_), 2.03 (s, 3H, -COCH_3_), 2.24 (s, 3H, -CH_3_), 2.25 (s, 3H, -CH_3_), 2.46 (s, 3H, -CH_3_), 3.30 (dd, *J* = 14.6, 7.5 Hz, 1H, H-6′a), 3.34 (dd, *J* = 14.6, 3.2 Hz, 1H, H-6′b), 3.77 (ddd, *J* = 9.6, 7.5, 3.2 Hz, 1H, H-5′), 4.98 (t, *J* = 9.6 Hz, 1H, H-4′), 5.03 (dd, *J* = 9.6, 8.3 Hz, 1H, H-2′), 5.18 (t, *J* = 9.6 Hz, 1H, H-3′), 5.59 (t, *J* = 8.3 Hz, 1H, H-1′), 13.34 (s, 1H, C(8)-OH), 13.46 (s, 1H, C(5)-OH). ^13^C NMR (125 MHz, CDCl_3_) δ: 12.4 (2×-CH_3_), 15.1 (-CH_3_), 20.3 (-COCH_3_), 20.5 (2×-COCH_3_), 20.6 (-COCH_3_), 34.5 (C-6′), 70.2 (C-2′), 70.8 (C-4′), 72.6 (C-3′), 75.4 (C-5′), 91.5 (C-1′), 109.1 (C-4a), 109.4 (C-8a), 140.4 (C-6), 140.8 (C-3), 141.1 (C-2), 146.7 (C-7), 168.6 (-COCH_3_), 169.2 (-COCH_3_), 169.5 (-COCH_3_), 170.1 (-COCH_3_), 171.3 (C-8), 171.6 (C-1, C-5), 171.9 (C-4). IR (CHCl_3_) *ν*_max_: 3705, 3609, 3026, 2944, 1760, 1600, 1559, 1429, 1396, 1373, 1340, 1302, 1250, 1219, 1192, 1121, 1077, 1039 cm^−1^. HRMS (ESI): *m*/*z* [M + Na]^+^ calcd for C_27_H_30_NaO_13_S: 617.1299; found: 617.1297.

*6-(1,2,3,4-Tetra-O-acetyl-6-deoxy-β-d-glucopyranos-6-ylthio)-7-ethyl-5,8-dihydroxy-2,3-dimethyl-1,4-naphthoquinone* (**SAB-17**). Dark red solid; yield 481 mg (79%); mp 179–182 °C; *R_f_* = 0.59 (A). ^1^H NMR (CDCl_3_, 500 MHz) δ: 1.15 (t, *J* = 7.6 Hz, 1H, -CH_2_CH_3_), 1.89 (s, 3H, -COCH_3_), 1.99 (s, 6H, 2 × -COCH_3_), 2.03 (s, 3H, -COCH_3_), 2.24 (s, 6H, 2 × -CH_3_), 3.02 (q, *J* = 7.6 Hz, 2H, -CH_2_CH_3_), 3.30 (dd, *J* = 14.5, 7.7 Hz, 1H, H-6′a), 3.34 (dd, *J* = 14.5, 3.0 Hz, 1H, H-6′b), 3.77 (ddd, *J* = 9.6, 7.7, 3.0 Hz, 1H, H-5′), 4.98 (t, *J* = 9.6 Hz, 1H, H-4′), 5.04 (dd, *J* = 9.6, 8.3 Hz, 1H, H-2′), 5.18 (t, *J* = 9.6 Hz, 1H, H-3′), 5.60 (t, *J* = 8.3 Hz, 1H, H-1′), 13.36 (s, 1H, C(8)-OH), 13.49 (s, 1H, C(5)-OH). ^13^C NMR (125 MHz, CDCl_3_) δ: 12.4 (2 × -CH_3_), 13.6 (-CH_2_CH_3_), 20.3 (-COCH_3_), 20.5 (2 × -COCH_3_), 20.6 (-COCH_3_), 22.4 (-CH_2_CH_3_), 34.5 (C-6′), 70.2 (C-2′), 70.9 (C-4′), 72.6 (C-3′), 75.3 (C-5′), 91.4 (C-1′), 109.2 (C-4a), 109.7 (C-8a), 139.7 (C-6), 140.9 (C-3), 141.3 (C-2), 152.1 (C-7), 168.5 (-COCH_3_), 169.2 (-COCH_3_), 169.5 (-COCH_3_), 170.0 (-COCH_3_), 170.8 (C-8), 171.7 (C-5), 171.9 (C-1), 172.1 (C-4). IR (CHCl_3_) *ν*_max_: 3668, 3601, 3028, 2937, 2875, 1760, 1600, 1556, 1456, 1372, 1338, 1303, 1282, 1249, 1194, 1124, 1076, 1039 cm^−1^. HRMS (ESI): *m*/*z* [M + Na]^+^ calcd for C_28_H_32_NaO_13_S: 631.1456; found: 631.1453.

*2-(1,2,3,4-Tetra-O-acetyl-6-deoxy-β-d-glucopyranos-6-ylthio)-3,6,7-trichloro-5,8-dihydroxy-1,4-naphthoquinone* (**SAB-19**). Purple solid; yield 420 mg (64%); mp 156–159 °C; *R_f_* = 0.48 (A). ^1^H NMR (CDCl_3_, 700 MHz) δ: 1.79 (s, 3H, -COCH_3_), 1.99 (s, 3H, -COCH_3_), 2.00 (s, 3H, -COCH_3_), 2.06 (s, 3H, -COCH_3_), 3.39 (dd, *J* = 14.7, 2.6 Hz, 1H, H-6′a), 3.66 (dd, *J* = 14.7, 8.0 Hz, 1H, H-6′b), 3.89 (ddd, *J* = 9.8, 8.0, 2.6 Hz, 1H, H-5′), 5.02 (t, *J* = 9.8 Hz, 1H, H-4′), 5.03 (dd, *J* = 9.4, 8.4 Hz, 1H, H-2′), 5.20 (t, *J* = 9.4 Hz, 1H, H-3′), 5.54 (t, *J* = 8.4 Hz, 1H, H-1′), 12.96 (s, 2H, C(5)-OH, C(8)-OH). ^13^C NMR (176 MHz, CDCl_3_) δ: 20.2 (-COCH_3_), 20.5 (2×-COCH_3_), 20.6 (-COCH_3_), 34.6 (C-6′), 70.0 (C-2′), 70.7 (C-4′), 72.5 (C-3′), 75.6 (C-5′), 91.7 (C-1′), 109.5 (C-4a), 109.7 (C-8a), 135.4 (C-6, C-7), 141.3 (C-3), 146.3 (C-2), 159.4 (C-8), 160.0 (C-5), 168.5 (-COCH_3_), 169.1 (-COCH_3_), 169.5 (-COCH_3_), 170.0 (-COCH_3_), 173.3 (C-4), 177.8 (C-1). IR (CHCl_3_) *ν*_max_: 3697, 3605, 3026, 2944, 1761, 1616, 1559, 1523, 1404, 1371, 1271, 1248, 1192, 1076, 1041 cm^−1^. HRMS (ESI): *m*/*z* [M + Na]^+^ calcd for C_24_H_21_Cl_3_NaO_13_S: 676.9660; found: 676.9655.

#### 4.1.3. General Procedure for Synthesis of the Acetylated Conjugates **SAB-3**, **-5**, and **-11** in DMSO Solution (Figure 1A)

The tetra-*O*-acetyl-6-mercaptoglucose (0.368 g, 1.01 mmol) and finely powdered potassium carbonate (0.415 g, 3.0 mmol) was added to a solution of correspondent quinone (1.0 mmol) in DMSO (20 mL). The mixture was stirred at room temperature until reaction completed (3 h, controlled by TLC), diluted with H_2_O (200 mL), acidified with HCl, and extracted with AcOEt (3 × 100 mL). The extract was washed with water (2 × 50 mL), dried with anhydrous Na_2_SO_4_, and concentrated in vacuo. The residue was purified by column chromatography (silica gel, hexane/acetone, 10:1) to give products **3, 5,** and **11**.

*6-(1,2,3,4-Tetra-O-acetyl-6-deoxy-β-d-glucopyranos-6-ylthio)-2,3,5,8-tetrahydroxy-7-methyl-1,4-naphthoquinone* (**SAB-3**). Red solid; yield 467 mg (78%); mp 207–211 °C; *R_f_* = 0.12 (A). ^1^H NMR (CDCl_3_, 500 MHz) δ: 1.99 (s, 3H, -COCH_3_), 2.00 (s, 6H, 2 × COCH_3_), 2.02 (s, 3H, -COCH_3_), 2.49 (s, 3H, C(7)-CH_3_), 3.16 (dd, *J* = 14.5, 8.0 Hz, 1H, H-6′a), 3.33 (dd, *J* = 14.5, 2.6 Hz, 1H, H-6′b), 3.76 (ddd, *J* = 9.4, 8.0, 2.6 Hz, 1H, H-5′), 5.00 (t, *J* = 9.4 Hz, 1H, H-4′), 5.06 (dd, *J* = 9.4, 8.3 Hz, 1H, H-2′), 5.19 (t, *J* = 9.4 Hz, 1H, H-3′), 5.62 (d, *J* = 8.3 Hz, 1H, H-1′), 6.82 (br.s, 2H, C(2)-OH, C(3)-OH), 12.24 (s, 1H, C(5)-OH), 12.70 (s, 1H, C(8)-OH). ^13^C NMR (CDCl_3_, 125 MHz) δ: 15.0 (C(7)-CH_3_), 20.5 (3×-COCH_3_), 20.6 (-COCH_3_), 34.7 (C-6′), 70.2 (C-2′), 70.9 (C-4′), 72.6 (C-3′), 74.9 (C-5′), 91.5 (C-1′), 106.2 (C-8a), 107.0 (C-4a), 136.3 (C-6), 137.8 (C-2 *), 138.0 (C-3 *), 144.3 (C-7), 156.5 (C-8), 157.9 (C-5), 168.7 (-COCH_3_), 169.2 (-COCH_3_), 169.5 (-COCH_3_), 170.1 (-COCH_3_), 182.4 (C-4), 182.7 (C-1). IR (CHCl_3_) *ν*_max_: 3432, 2944, 1760, 1693, 1626, 1594, 1556, 1419, 1373, 1310 cm^−1^. HRMS (ESI): *m*/*z* [M + Na]^+^ calcd for C_25_H_26_O_15_SNa 621.0885; found: 621.0882. *—The assignment of signal is ambiguous.

*7-(1,2,3,4-Tetra-O-acetyl-6-deoxy-β-d-glucopyranos-6-ylthio)-2,5,8-trihydroxy-6-methoxy-1,4-naphthoquinone* (**SAB-5**). Red solid; yield 461 mg (77%); mp 109–112 °C; *R_f_* = 0.20 (A). ^1^H NMR (CDCl_3_, 500 MHz) δ: 1.90 (s, 3H, -COCH_3_), 1.89 (s, 3H, -COCH_3_), 1.99 (s, 3H, -COCH_3_), 2.03 (s, 3H, -COCH_3_), 3.13 (dd, *J* = 14.5, 2.8 Hz, 1H, H-6′a), 3.29 (dd, *J* = 14.5, 7.7 Hz, 1H, H-6′b), 3.83 (ddd, *J* = 9.5, 7.7, 2.8 Hz, 1H, H-5′), 4.22 (s, 3H, C(6)-OCH_3_), 5.02 (t, *J* = 9.5 Hz, 1H, H-4′), 5.04 (dd, *J* = 9.5, 8.3 Hz, 1H, H-2′), 5.19 (t, *J* = 9.5 Hz, 1H, H-3′), 5.59 (d, *J* = 8.3 Hz, 1H, H-1′), 6.42 (s, 1H, H-3), 7.39 (br s, 1H, C(2)-OH), 12.65 (s, 1H, C(8)-OH), 13.17 (s, 1H, C(5)-OH). ^13^C NMR (CDCl_3_, 125 MHz) δ: 20.4 (-COCH_3_), 20.5 (2×-COCH_3_), 20.6 (-COCH_3_), 33.8 (C-6′), 61.9 (C(6)-OCH_3_), 70.2 (C-2′), 70.9 (C-4′), 72.6 (C-3′), 75.6 (C-5′), 91.4 (C-1′), 107.6 (C-8a), 109.0 (C-4a), 110.2 (C-3), 126.2 (C-7), 157.0 (C-2), 160.3 (C-6), 160.5 (C-5), 168.3 (C-8), 168.5 (-COCH_3_), 169.2 (-COCH_3_), 169.5 (-COCH_3_), 170.1 (-COCH_3_), 170.4 (C-1), 180.4 (C-4). IR (CHCl_3_) *ν*_max_: 3514, 3404, 2945, 1760, 1659, 1604, 1553, 1445, 1397, 1370, 1349 cm^−1^. HRMS (ESI): *m*/*z* [M + Na]^+^ calcd for C_25_H_26_O_15_SNa 621.0885; found: 621.0881.

*7-(1,2,3,4-Tetra-O-acetyl-6-deoxy-β-d-glucopyranos-6-ylthio)-3-ethyl-2,5,8-trihydroxy-6-methoxy-1,4-naphthoquinone* (**SAB-11**). Red solid; yield 501 mg (80%); mp 167–171 °C; *R_f_* = 0.33 (A). ^1^H NMR (CDCl_3_, 700 MHz) δ: 1.13 (t, *J* = 7.5, 3H, -CH_2_CH_3_), 1.93 (s, 3H, -COCH_3_), 1.98 (s, 3H, -COCH_3_), 1.99 (s, 3H, -COCH_3_), 2.02 (s, 3H, -COCH_3_), 2.62 (q, *J* = 7.5, 2H, -CH_2_CH_3_), 4.17 (s, 3H, C(6)-OCH_3_), 3.16 (dd, *J* = 14.2, 2.8 Hz, 1H, H-6′a), 3.24 (dd, *J* = 14.2, 7.7 Hz, 1H, H-6′b), 3.81 (ddd, *J* = 9.5, 7.7, 2.8 Hz, 1H, H-5′), 5.02 (t, *J* = 9.5 Hz, 1H, H-4′), 5.04 (dd, *J* = 9.5, 8.2 Hz, 1H, H-2′), 5.18 (t, *J* = 9.5 Hz, 1H, H-3′), 5.61 (d, *J* = 8.2 Hz, 1H, H-1′), 7.41 (br s, 1H, C(2)-OH), 12.56 (s, 1H, C(8)-OH), 13.47 (s, 1H, C(5)-OH). ^13^C NMR (176 MHz, CDCl_3_) δ: 12.6 (-CH_2_CH_3_), 16.2 (-CH_2_CH_3_), 20.4 (-COCH_3_), 20.5 (2×-COCH_3_), 20.6 (-COCH_3_), 34.1 (C-6′), 61.7 (C(6)-OCH_3_), 70.3 (C-2′), 70.9 (C-4′), 72.6 (C-3′), 75.4 (C-5′), 91.4 (C-1′), 106.2 (C-8a), 110.0 (C-4a), 125.3 (C-7), 126.4 (C-3), 153.7 (C-2), 155.3 (C-5), 159.7 (C-6), 162.6 (C-8), 168.5 (-COCH_3_), 169.2 (-COCH_3_), 169.5 (-COCH_3_), 170.0 (-COCH_3_), 176.8 (C-1), 185.2 (C-4). IR (CHCl_3_) *ν*_max_: 3408, 2976, 2941, 2878, 1760, 1598, 1555, 1460 cm^−1^. HRMS (EI): *m/z* [M + Na]^+^ calcd for C_27_H_30_O_15_SNa: 649.1198; found: 649.1192.

#### 4.1.4. Procedure for Synthesis of Naphthazarin Derivative SAB-21 (Figure 1B)

Tetra-*O*-acetyl-6-mercaptoglucose (190 mg, 0.52 mmol) was added to the refluxing solution of naphthazarin (190 mg, 1.0 mmol) in 30 mL of EtOH during 30 min. The reaction mixture was refluxed for 1.5 h, concentrated in vacuo. The residue was purified by column chromatography (silica gel, hexane/acetone, 10:1) to give the product **21**. Naphthazarin recovery was 44% (83 mg).

*2-(1,2,3,4-Tetra-O-acetyl-6-deoxy-β-d-glucopyranos-6-ylthio)-5,8-dihydroxy-1,4-naphthoquinone* (**SAB-21**). Purple solid; yield 167 mg (54%); mp 207–210 °C; *R_f_* = 0.38 (A). ^1^H NMR (CDCl_3_, 500 MHz) δ: 2.02 (s, 6H, -COCH_3_), 2.03 (s, 3H, -COCH_3_), 2.08 (s, 3H, -COCH_3_), 2.13 (s, 3H, -COCH_3_), 3.02 (dd, *J* = 14.1, 6.9 Hz, 1H, H-6′a), 3.10 (dd, *J* = 14.1, 3.2 Hz, 1H, H-6′b), 3.96 (ddd, *J* = 9.5, 6.9, 3.2 Hz, 1H, H-5′), 5.11 (t, *J* = 9.5 Hz, 1H, H-4′), 5.14 (dd, *J* = 9.5, 8.4 Hz, 1H, H-2′), 5.26 (t, *J* = 9.4 Hz, 1H, H-3′), 5.71 (t, *J* = 8.4 Hz, 1H, H-1′), 6.68 (s, 1H, H-3), 7.21 (dd, *J* = 9.4 Hz, 1H, H-7), 7.27 (dd, *J* = 9.4 Hz, 1H, H-6), 12.11 (s, 1H, C(8)-OH), 12.57 (s, 1H, C(5)-OH). ^13^C NMR (125 MHz, CDCl_3_) δ: 20.5 (2×-COCH_3_), 20.6 (-COCH_3_), 20.7 (-COCH_3_), 31.9 (C-6′), 70.1 (C-2′), 71.0 (C-4′), 72.5 (C-3′), 73.3 (C-5′), 91.6 (C-1′), 111.1 (C-4a), 111.5 (C-8a), 128.1 (C-3), 128.8 (C-7), 130.8 (C-6), 154.2 (C-2), 158.5 (C-5), 159.4 (C-8), 168.8 (-COCH_3_), 169.1 (-COCH_3_), 169.6 (-COCH_3_), 170.0 (-COCH_3_), 183.6 (C-1), 183.9 (C-4). IR (CHCl_3_) *ν*_max_: 3695, 3600, 3054, 2928, 1761, 1607, 1577, 1556, 1455, 1409, 1371, 1338, 1240, 1221, 1193, 1109, 1077, 1039 cm^−1^. HRMS (ESI): *m*/*z* [M + Na]^+^ calcd for C_24_H_24_NaO_13_S: 575.0830; found: 575.0829.

#### 4.1.5. General Procedure for Synthesis of Juglone Derivatives 23 and 25 (Figure 1B)

Tetra-*O*-acetyl-6-mercaptoglucose (0.367 g, 1.01 mmol) was added to a suspension of juglone or juglone acetate (1.0 mmol) in EtOH (65 mL). The reaction mixture was stirred at room temperature for 12 h and concentrated in vacuo. The residue was purified by column chromatography (silica gel, hexane/acetone, 10:1) to give the products **23** and **25**.

*2-(1,2,3,4-Tetra-O-acetyl-6-deoxy-β-d-glucopyranos-6-ylthio)-8-hydroxy-1,4-naphthoquinone* (**SAB-23**). Yellow solid; yield 349 mg (65%); mp 188–191 °C; *R_f_* = 0.39 (A). ^1^H NMR (CDCl_3_, 500 MHz) δ: 2.02 (s, 3H, -COCH_3_), 2.03 (s, 3H, -COCH_3_), 2.07 (s, 3H, -COCH_3_), 2.12 (s, 3H, -COCH_3_), 3.00 (dd, *J* = 14.0, 7.0 Hz, 1H, H-6′a), 3.07 (dd, *J* = 14.0, 3.2 Hz, 1H, H-6′b), 3.95 (ddd, *J* = 9.6, 7.0, 3.2 Hz, 1H, H-5′), 5.10 (d, *J* = 9.6 Hz, 1H, H-4′), 5.14 (dd, *J* = 9.5, 8.3 Hz, 1H, H-2′), 5.26 (d, *J* = 9.5 Hz, 1H, H-3′), 5.71 (d, *J* = 8.3 Hz, 1H, H-1′), 6.64 (s, 1H, H-3), 7.23 (dd, *J* = 8.0, 1.5 Hz, 1H, H-7), 7.60 (dd, *J* = 7.4, 1.5 Hz, 1H, H-5), 7.62 (dd, *J* = 8.0, 7.4 Hz, 1H, H-6), 11.65 (s, 3H, C(8)-OH). ^13^C NMR (125 MHz, CDCl_3_) δ: 20.5 (2 × -COCH_3_), 20.6 (-COCH_3_), 20.7 (-COCH_3_), 31.8 (C-6′), 70.1 (C-2′), 71.0 (C-4′), 72.6 (C-3′), 73.3 (C-5′), 91.6 (C-1′), 114.7 (8a), 119.3 (C-5), 123.9 (C-7), 128.7 (C-3), 132.0 (C-4a), 137.1 (C-6), 153.3 (C-2), 161.9 (C-8), 168.8 (-COCH_3_), 169.1 (-COCH_3_), 169.6 (-COCH_3_), 170.0 (-COCH_3_), 180.8 (C-4), 186.8 (C-1). IR (CHCl_3_) *ν*_max_: 3700, 3600, 2942, 1761, 1634, 1601, 1562, 1457, 1427, 1369, 1273, 1246, 1194, 1169, 1140, 1078, 1039 cm^−1^. HRMS (ESI): *m*/*z* [M + Na]^+^ calcd for C_24_H_24_NaO_12_S: 536.0881; found: 536.0880.

*2-(1,2,3,4-Tetra-O-acetyl-6-deoxy-β-d-glucopyranos-6-ylthio)-5-acetoxy-1,4-naphthoquinone* (**SAB-25**). Yellow solid; yield 353 mg (61%); mp 172–175 °C; *R_f_* = 0.30 (A). ^1^H NMR (CDCl_3_, 500 MHz) δ: 2.02 (s, 6H, 2 × -COCH_3_), 2.06 (s, 3H, -COCH_3_), 2.11 (s, 3H, -COCH_3_), 2.43 (s, 3H, -COCH_3_′), 2.97 (dd, *J* = 14.1, 7.1 Hz, 1H, H-6′a), 3.03 (dd, *J* = 14.1, 3.2 Hz, 1H, H-6′b), 3.93 (ddd, *J* = 9.6, 7.1, 3.2 Hz, 1H, H-5′), 5.09 (d, *J* = 9.6 Hz, 1H, H-4′), 5.12 (dd, *J* = 9.5, 8.3 Hz, 1H, H-2′), 5.24 (d, *J* = 9.3 Hz, 1H, H-3′), 5.70 (d, *J* = 8.3 Hz, 1H, H-1′), 6.54 (s, 1H, H-3), 7.37 (dd, *J* = 8.0, 1.2 Hz, 1H, H-6), 7.71 (dd, *J* = 8.0, 7.8 Hz, 1H, H-7), 8.06 (dd, *J* = 7.8, 1.3 Hz, 1H, H-8). ^13^C NMR (125 MHz, CDCl_3_) δ: 20.5 (2 × -COCH_3_), 20.6 (-COCH_3_), 20.7 (-COCH_3_), 21.1 (-COCH_3_′), 31.7 (C-6′), 70.1 (C-2′), 71.0 (C-4′), 72.6 (C-3′), 73.4 (C-5′), 91.5 (C-1′), 123.2 (4a), 125.5 (C-8), 129.3 (C-3), 130.2 (C-6), 133.6 (C-8a), 134.2 (C-7), 149.6 (C-5), 152.2 (C-2), 168.8 (-COCH_3_), 169.1 (-COCH_3_), 169.3 (-COCH_3_), 169.6 (-COCH_3_), 170.0 (-COCH_3_), 180.2 (C-4), 181.2 (C-1). IR (CHCl_3_) *ν*_max_: 3700, 3600, 3016, 2942, 1761, 1671, 1650, 1600, 1566, 1541, 1522, 1457, 1370, 1337, 1247, 1193, 1106, 1078, 1039 cm^−1^. HRMS (ESI): *m*/*z* [M + Na]^+^ calcd for C_26_H_26_NaO_13_S: 601.0986; found: 601.0982.

#### 4.1.6. General Procedure for Synthesis of Deacetylated Conjugates **SAB-2**, **-4**, **-6**, **-8**, **-10**, **-12**, **-14**, **-16**, **-18**, **-20**, **-22**, **-24**, and **-26** (Figure 1C, Saponification of the Acetylated Conjugates)

A solution of MeONa (0.9 mL of a 1.06 N) in MeOH was added to a stirring suspension of appropriate acetylated conjugate (0.3 mmol) in anhydrous MeOH (20 mL). The reaction mixture was stirred for 30 min, neutralized with HCl, concentrated in vacuo. The residue was purified by preparative TLC (silica gel, benzene/ethyl acetate/methanol 7:4:2 (v/v)) to give the products.

*2-(6-Deoxy-d-glucopyranos-6-ylthio)-5,8-dihydroxy-6,7-dimethyl-1,4-naphthoquinone* (**SAB-2**). Red solid; yield 93 mg (75%); *R_f_* = 0.70 (B). *α*-anomer: ^1^H NMR (500 MHz, DMSO-d_6_) δ: 2.14 (s, 3H, C(7)-CH_3_), 2.15 (s, 3H, C(6)-CH_3_), 3.02 (dd, *J =* 13.3, 8.4 Hz, 1H, H-6′a), 3.09 (dd, *J =* 9.9, 8.7Hz, 1H, H-4′), 3.19 (dd, *J =* 9.6, 3.6 Hz, 1H, H-2′), 3.36 (dd, *J =* 13.3, 2.4 Hz, 1H, H-6′b), 3.45 (dd, *J =* 9.6, 8.7 Hz, 1H, H-3′), 3.84 (ddd, *J =* 9.9, 8.4, 2.4 Hz, 1H, H-5′), 4.93 (d, *J =* 3.6 Hz, 1H, H-1′), 6.90 (s, 1H, H-3), 12.83 (s, 1H, C(8)-OH), 13.10 (s, 1H, C(5)-OH). ^13^C NMR (125 MHz, DMSO-d_6_) δ: 12.1 (C(7)-CH_3_), 12.3 (C(6)-CH_3_), 32.8 (C-6′), 69.3 (C-5′), 72.3 (C-2′), 72.7 (C-3′), 73.7 (C-4′), 92.6 (C-1′), 107.9 (C-4a), 109.0 (C-8a), 124.7 (C-3), 138.7 (C-7), 141.1 (C-6), 151.3 (C-2), 166.3 (C-5), 167.5 (C-8), 173.4 (C-1), 174.7 (C-4). *β*-anomer: ^1^H NMR (500 MHz, DMSO-d_6_) δ: 2.14 (s, 3H, C(7)-CH_3_), 2.15 (s, 3H, C(6)-CH_3_), 2.95 (dd, *J =* 8.8, 7.7 Hz, 1H, H-2′), 3.03 (dd, *J =* 14.0, 8.0 Hz, 1H, H-6′a), 3.11 (t, *J =* 8.8 Hz, 1H, H-4′), 3.17 (t, *J =* 8.8 Hz, 1H, H-3′), 3.38 (dd, *J =* 14.0, 2.5 Hz, 1H, H-6′b), 3.40 (ddd, *J =* 8.8, 8.0, 2.5 Hz, 1H, H-5′), 4.34 (d, *J =* 7.7 Hz, 1H, H-1′), 6.89 (s, 1H, H-3), 12.82 (s, 1H, C(8)-OH), 13.10 (s, 1H, C(5)-OH). ^13^C NMR (125 MHz, DMSO-d_6_) δ: 12.1 (C(7)-CH_3_), 12.3 (C(6)-CH_3_), 32.6 (C-6′), 73.1 (C-4′), 73.6 (C-5′), 74.8 (C-2′), 76.2 (C-3′), 97.1 (C-1′), 107.9 (C-4a), 109.0 (C-8a), 124.8 (C-3), 138.7 (C-7), 141.1 (C-6), 151.1 (C-2), 166.1 (C-5), 167.3 (C-8), 173.6 (C-1), 174.9 (C-4). HRMS (ESI): *m*/*z* [M + Na]^+^ calcd for C_18_H_20_O_9_SNa: 435.0720; found: 435.0720.

*6-(6-Deoxy-d-glucopyranos-6-ylthio)-2,3,5,8-tetrahydroxy-7-methyl-1,4-naphthoquinone* (**SAB-4**). Red solid; yield 101 mg (78%); *R_f_* = 0.57 (B). *α*-anomer: ^1^H NMR (DMSO-d_6_, 300 MHz) δ: 2.43 (s, 3H, C(7)-CH_3_), 3.00 (dd, *J =* 13.7, 8.0 Hz, 1H, H-6′a), 3.08 (t, *J =* 9.2Hz, 1H, H-4′), 3.14 (dd, *J =* 9.3, 3.4Hz, 1H, H-2′), 3.38 (t, *J =* 9.3 Hz, 1H, H-3′), 3.39 (dd, *J =* 13.7, 2.6 Hz, 1H, H-6′b), 3.71 (ddd, *J =* 9.3, 8.0, 2.6 Hz, 1H, H-5′), 4.83 (d, *J =* 3.4 Hz, 1H, H-1′), 10.53 (br s, 2H, C(2)-OH, C(3)-OH), 12.88 (s, 1H, C(5)-OH), 13.20 (s, 1H, C(8)-OH). ^13^C NMR (DMSO-d_6_, 75 MHz) δ: 14.9 (C(7)-CH_3_), 36.6 (C-6′), 70.8 (C-5′), 72.4 (C-2′), 72.8 (C-3′), 73.2 (C-4′), 92.4 (C-1′), 106.8 (C-8a), 107.7 (C-4a), 136.3 (C-6), 141.3 (C-2*), 141.4 (C-3*), 141.9 (C-7), 154.9 (C-8), 157.1 (C-5), 183.2 (C-4), 183.5 (C-1). *β*-anomer: ^1^H NMR (DMSO-d_6_, 300 MHz) δ: 2.43 (s, 3H, C(7)-CH_3_), 2.89 (dd, *J =* 8.8, 7.7Hz, 1H, H-2′), 2.95 (dd, *J =* 13.3, 8.2 Hz, 1H, H-6′a), 3.02 (t, *J =* 8.8 Hz, 1H, H-4′), 3.07 (t, *J =* 8.8 Hz, 1H, H-3′), 3.18 (ddd, *J =* 8.8, 8.2, 2.2 Hz, 1H, H-5′), 3.35 (dd, *J =* 13.3, 2.2 Hz, 1H, H-6′b), 4.24 (d, *J =* 7.7 Hz, 1H, H-1′), 10.53 (br s, 2H, C(2)-OH, C(3)-OH), 12.88 (s, 1H, C(5)-OH), 13.20 (s, 1H, C(8)-OH).^13^C NMR (DMSO-d_6_, 75 MHz) δ: 14.9 (C(7)-CH_3_), 36.8 (C-6′), 72.8 (C-4′), 74.9 (C-2′), 75.3 (C-5′), 76.4 (C-3′), 97.1 (C-1′), 106.9 (C-8a), 107.8 (C-4a), 135.9 (C-6), 141.3 (C-2*), 141.4 (C-3*), 142.1 (C-7), 154.9 (C-8), 157.1 (C-5), 183.1 (C-4), 183.3 (C-1). HRMS (ESI): *m*/*z* [M + Na]^+^ calcd for C_17_H_18_O_11_SNa: 453.0462; found: 453.0459. *–The assignment of signal is ambiguous.

*7-(6-Deoxy-d-glucopyranos-6-ylthio)-2,5,8-trihydroxy-6-methoxy-1,4-naphthoquinone* (**SAB-6**). Red solid; yield 97 mg (75%), *R_f_* = 0.81(B). *α*-anomer: ^1^H NMR (500 MHz, DMSO-d_6_) δ: 3.06 (t, *J* = 9.2 Hz, 1H, H-4′), 3.12 (dd, *J* = 13.0, 8.2 Hz, 1H, H-6′a), 3.13 (dd, *J* = 9.2, 3.6 Hz, 1H, H-2′), 3.39 (t, *J* = 9.2 Hz, 1H, H-3′), 3.45 (dd, *J* = 13.0, 2.6 Hz, 1H, H-6′b), 3.77 (ddd, *J* = 9.2, 8.2, 2.6 Hz, 1H, H-5′), 4.06 (s, 3H, C(6)-OCH_3_), 4.84 (d, *J* = 3.6 Hz, 1H, H-1′), 6.39 (s, 1H, H-3), 11.90 (br s, 1H, C(2)-OH), 12.95 (s, 1H, C(8)-OH), 13.08 (s, 1H, C(5)-OH).^13^C NMR (125 MHz, DMSO-d_6_) δ: 35.7 (C-6′), 61.2 (C(6)-OCH_3_), 70.8 (C-5′), 72.3 (C-2′), 72.8 (C-3′), 73.1 (C-4′), 92.4 (C-1′), 107.1 (C-8a), 109.4 (C-4a), 109.8 (C-3), 130.2 (C-7), 158.9 (C-6), 159.2 (C-2), 165.0 (C-8), 165.7 (C-5), 173.4 (C-1), 174.8 (C-4). *β*-anomer: ^1^H NMR (500 MHz, DMSO-d_6_) δ: 2.89 (dd, *J* = 9.0, 7.7 Hz, 1H, H-2′), 3.06 (t, *J* = 9.0 Hz, 1H, H-4′), 3.09 (t, *J* = 9.0 Hz, 1H, H-3′), 3.11 (dd, *J* = 13.3, 8.0 Hz, 1H, H-6′a), 3.28 (ddd, *J* = 9.0, 8.0, 2.4 Hz, 1H, H-5′), 3.46 (dd, *J* = 13.3, 2.4 Hz, 1H, H-6′b), 4.07 (s, 3H, C(6)-OCH_3_), 4.26 (d, *J* = 7.7 Hz, 1H, H-1′), 6.38 (s, 1H, H-3), 11.90 (br s, 1H, C(2)-OH), 12.96 (s, 1H, C(8)-OH), 13.08 (s, 1H, C(5)-OH). ^13^C NMR (125 MHz, DMSO-d_6_) δ: 35.5 (C-6′), 61.3 (C(6)-OCH_3_), 72.7 (C-4′), 74.9 (C-2′), 75.4 (C-5′), 76.4 (C-3′), 97.0 (C-1′), 107.3 (C-8a), 109.5 (C-4a), 109.8 (C-3), 129.7 (C-7), 159.0 (C-6), 159.2 (C-2), 165.3 (C-8), 165.5 (C-5), 173.2 (C-1), 174.0 (C-4). HRMS (ESI): *m*/*z* [M + Na]^+^ calcd for C_17_H_18_O_11_SNa: 453.0462; found: 453.0458.

*2-(6-Deoxy-d-glucopyranos-6-ylthio)-5-hydroxy-3-methyl-1,4-naphthoquinone* (**SAB-8**). Red solid; yield 86 mg (75%); *R_f_* = 0.77 (B). *α*-anomer: ^1^H NMR (700 MHz, DMSO-d_6_) δ: 2.24 (s, 3H, C(3)-CH_3_), 3.03 (dd, *J* = 9.2, 3.6 Hz, 1H, H-2′), 3.04 (t, *J* = 9.2 Hz, 1H, H-4′), 3.26 (dd, *J* = 13.6, 8.0 Hz, 1H, H-6′a), 3.36 (t, *J* = 9.2 Hz, 1H, H-3′), 3.48 (dd, *J* = 13.6, 2.5 Hz, 1H, H-6′b), 3.74 (ddd, *J* = 9.2, 8.0, 2.5 Hz, 1H, H-5′), 4.81 (d, *J* = 3.6 Hz, 1H, H-1′), 7.29 (d, *J* = 8.0, 1H, H-6), 7.50 (d, *J* = 8.0, 1H, H-8), 7.69 (t, *J* = 8.0, 1H, H-7), 11.98 (s, 1H, C(5)-OH). ^13^C NMR (175 MHz, DMSO-d_6_) δ: 14.4 (C(3)-CH_3_), 35.7 (C-6′), 71.3 (C-5′), 72.5 (C-2′), 72.6 (C-3′), 72.8 (C-4′), 92.3 (C-1′), 114.8 (C-4a), 119.1 (C-8), 123.4 (C-6), 133.1 (C-8a), 136.1 (C-7), 144.5 (C-3), 148.7 (C-2), 160.2 (C-5), 180.1 (C-1), 186.8 (C-4). *β*-anomer: ^1^H NMR (700 MHz, DMSO-d_6_) δ: 2.26 (s, 3H, C(3)-CH_3_), 2.84 (dd, *J* = 8.2, 7.7 Hz, 1H, H-2′), 3.04 (t, *J* = 8.2 Hz, 1H, H-4′), 3.07 (t, *J* = 8.2 Hz, 1H, H-3′), 3.25 (dd, *J* = 13.3, 7.5 Hz, 1H, H-6′a), 3.28 (ddd, *J* = 8.2, 7.5, 2.1 Hz, 1H, H-5′), 3.52 (dd, *J* = 13.3, 2.1 Hz, 1H, H-6′b), 4.26 (d, *J* = 7.7 Hz, 1H, H-1′), 7.28 (d, *J* = 8.0, 1H, H-6), 7.51 (d, *J* = 8.0, 1H, H-8), 7.71 (t, *J* = 8.0, 1H, H-7), 11.99 (s, 1H, C(5)-OH).^13^C NMR (175 MHz, DMSO-d_6_) δ: 14.5 (C(3)-CH_3_), 35.8 (C-6′), 72.4 (C-4′), 74.8 (C-2′), 75.4 (C-5′), 76.2 (C-3′), 97.0 (C-1′), 114.9 (C-4a), 119.2 (C-8), 123.5 (C-6), 133.0 (C-8a), 136.1 (C-7), 145.2 (C-3), 149.3 (C-2), 160.2 (C-5), 180.1 (C-1), 186.9 (C-4). HRMS (ESI): *m*/*z* [M + Na]^+^ calcd for C_17_H_18_O_8_SNa: 405.0615; found: 405.0618.

*2,3-Di(6-deoxy-d-glucopyranos-6-ylthio)-5,8-dihydroxy-6,7-dimethyl-1,4-naphthoquinone* (**SAB-10**). Dark red solid; yield 142 mg (78%); R*_f_* = 0.04 (B). ^1^H NMR (500 MHz, DMSO-d_6_) δ: α-anomer: 2.13 (s, 6H, (C(6,7)-CH_3_), 3.06 (t, *J =* 9.1 Hz, 2H, H-4′), 3.11 (dd, *J =* 13.2, 8.4 Hz, 2H, H-6a′), 3.12 (dd, *J =* 9.1, 3.4 Hz, 2H, H-2′), 3.39 (dd, *J =* 9.1 Hz, 2H, H-3′), 3.44 (dd, *J =* 13.2, 2.3 Hz, 2H, H-6b′), 3.77 (ddd, *J =* 9.1, 8.4, 2.3 Hz, 2H, H-5′), 4.77 (d, *J =* 3.4 Hz, 2H, H-1′), 13.08 (br s, 2H, C(5,8)-OH); β-anomer: 2.13 (s, 6H, (C(6,7)-CH_3_), 2.89 (dd, *J =* 8.2, 7.7Hz, 2H, H-2′), 3.05 (t, *J =* 8.2 Hz, 2H, H-4′), 3.08 (dd, *J =* 13.3, 7.7 Hz, 2H, H-6a′), 3.09 (dd, *J =* 8.2 Hz, 2H, H-3′), 3.27 (ddd, *J =* 8.2, 7.7, 2.0 Hz, 2H,H-5′), 3.45 (dd, *J =* 13.3, 2.0 Hz, 2H,H-6b′), 4.26 (d, *J =* 7.7 Hz, 2H,H-1′), 13.08 (br s, 2H, C(5,8)-OH); ^13^C NMR (125 MHz, DMSO-d_6_) δ: carbohydrate moiety: α-anomer: 35.9 (C-6′), 70.8 (C-5′), 72.3 (C-2′), 72.8 (C-3′), 73.2 (C-4′), 92.4 (C-1′), β-anomer: 35.7 (C-6′), 72.7 (C-4′), 74.9 (C-2′), 75.4 (C-5′), 76.4 (C-3′), 97.0 (C-1′); naphthoquinone moiety: 12.5 ((C-6,7)-CH_3_), 109.1 (C-4a,8a), 138.6 (C-2,3), 147.3 (C-6,7), 161.0 (C-5,8), 177.5 (C-4,8); HRMS (ESI): *m*/*z* [M + Na]^+^ calcd for C_24_H_30_O_14_S_2_Na: 629.0969; found: 629.0976.

*7-(6-Deoxy-d-glucopyranos-6-ylthio)-3-ethyl-2,5,8-trihydroxy-6-methoxy-1,4-naphthoquinone* (**SAB-12**). Red solid; yield 113 mg (82%); *R_f_* = 0.26 (B). *α*-anomer: ^1^H NMR (500 MHz, DMSO-d_6_) δ: 1.03 (t, *J =* 7.5, 3H, -CH_2_CH_3_), 2.49 (q, 2H, -CH_2_CH_3_), 3.06 (t, *J =* 9.1 Hz, 1H, H-4′), 3.11 (dd, *J =* 13.2, 8.4 Hz, 1H, H-6′a), 3.12 (dd, *J =* 9.1, 3.4Hz, 1H, H-2′), 3.39 (dd, *J =* 9.1 Hz, 1H, H-3′), 3.44 (dd, *J =* 13.2, 2.3 Hz, 1H, H-6′b), 3.77 (ddd, *J =* 9.1, 8.4, 2.3 Hz, 1H, H-5′), 4.02 (s, 3H,C(6)-OCH_3_), 4.84 (d, *J =* 3.4 Hz, 1H, H-1′), 11.35 (br s, 1H, C(2)-OH), 12.92 (s, 1H, C(8)-OH), 13.51 (s, 1H, C(5)-OH). ^13^C NMR (125 MHz, DMSO-d_6_) δ: 12.6 (-CH_2_CH_3_), 15.7 (-CH_2_CH_3_), 35.9 (C-6′), 61.0 (C(6)-OCH_3_), 70.8 (C-5′), 72.3 (C-2′), 72.8 (C-3′), 73.2 (C-4′), 92.4 (C-1′), 107.1 (C-8a), 108.9 (C-4a), 125.6 (C-3), 128.4 (C-7), 156.1 (C-2), 157.3 (C-5), 158.1 (C-6), 164.8 (C-8), 174.3 (C-1), 181.5 (C-4). *β*-anomer: ^1^H NMR (500 MHz, DMSO-d_6_) δ: 1.03 (t, *J =* 7.5, 3H, -CH_2_CH_3_), 2.49 (q, 2H, -CH_2_CH_3_), 2.89 (dd, *J =* 8.2, 7.7Hz, 1H, H-2′), 3.05 (t, *J =* 8.2 Hz, 1H, H-4′), 3.08 (dd, *J =* 13.3, 7.7 Hz, 1H, H-6′a), 3.09 (dd, *J =* 8.2 Hz, 1H, H-3′), 3.27 (ddd, *J =* 8.2, 7.7, 2.0 Hz, 1H, H-5′), 3.45 (dd, *J =* 13.3, 2.0 Hz, 1H, H-6′b), 4.02 (s, 3H,C(6)-OCH_3_), 4.26 (d, *J =* 7.7 Hz, 1H, H-1′), 11.35 (br s, 1H, C(2)-OH), 12.92 (s, 1H, C(8)-OH), 13.51 (s, 1H, C(5)-OH).^13^C NMR (125 MHz, DMSO-d_6_) δ: 12.6 (-CH_2_CH_3_), 15.7 (-CH_2_CH_3_), 35.7 (C-6′), 61.1 (C(6)-OCH_3_), 72.7 (C-4′), 74.9 (C-2′), 75.4 (C-5′), 76.4 (C-3′), 97.1 (C-1′), 107.2 (C-8a), 108.7 (C-4a), 125.6 (C-3), 127.9 (C-7), 156.1 (C-2), 157.4 (C-5), 158.2 (C-6), 164.6 (C-8), 174.5 (C-1), 181.7 (C-4). HRMS (ESI): *m*/*z* [M + Na]^+^ calcd for C_19_H_22_O_11_SNa: 481.0775; found: 481.0778.

*6-(6-Deoxy-d-glucopyranos-6-ylthio)-5,8-dihydroxy-7-methyl-2,3-dimethoxy-1,4-naphthoquinone* (**SAB-14**). Purple solid; yield 85 mg (62%); *R_f_* = 0.46 (B). *α*-anomer: ^1^H NMR (500 MHz, DMSO-d_6_) δ: 2.43 (s, 3H,С(7)-СH_3_), 3.04 (m, 1H, H-4′), 3.06 (dd, *J =* 13.6, 7.6 Hz, 1H, H-6′a), 3.11 (m, 1H, H-2′), 3.36 (m, 1H, H-3′),3.42 (dd, *J =* 13.6, 2.3Hz, 1H, H-6′b), 3.70 (m, 1H, H-5′), 4.01 (s, 3H,-OСH_3_), 4.02 (s, 3H,-OСH_3_), 4.48 (d, *J =* 6.6Hz, 1H, C(2′)-OH), 4.68 (d, *J =* 4.8Hz, 1H, C(3′)-OH), 4.81 (t, *J =* 4.8 Hz, 1H, H-1′), 4.94 (d, *J =* 5.7 Hz, 1H, C(4′)-OH), 6.27 (d, *J =* 4.8Hz, 1H, C(1′)-OH), 12.92 (s, 1H, C(8)-OH), 13.21 (s, 1H, C(5)-OH). ^13^C NMR (125 MHz, DMSO-d_6_) δ:15.0 (С(7)-СH_3_), 36.8 (С-6′), 61.4 (2× -OСH_3_), 70.8 (C-5′), 72.3 (C-2′), 72.8 (C-3′), 73.1 (C-4′), 92.4 (C-1′), 107.8 (C-4a), 108.5 (C-8a), 138.2 (C-6), 142.9 (C-7), 147.8 (C-3), 147.9 (C-2), 158.8 (C-8), 160.6 (C-5), 180.5 (C-1), 180.7 (C-4). *β*-anomer:^1^H NMR (500 MHz, DMSO-d_6_) δ: 2.43 (s, 3H, С(7)-СH_3_), 2.88 (m, 1H, H-2′), 3.04 (m, 3H,H-4′,H-3′, H-6′a), 3.20 (m, 1H, H-5′), 3.41 (dd, *J =* 13.5, 2.1 Hz, 1H, H-6′b), 4.01 (s, 3H,-OСH_3_), 4.02 (s, 3H,-OСH_3_), 4.23 (dd, *J =* 7.6, 6.4 Hz, 1H, H-1′), 4.84 (d, *J =* 4.8 Hz, 1H, C(2′)-OH), 4.86 (d, *J =* 4.4 Hz, 1H, C(3′)-OH), 4.99 (d, *J =* 5.0 Hz, 1H, C(4′-OH), 6.56 (d, *J =* 6.8 Hz, 1H, C(1′)-OH), 12.92 (s, 1H, C(8)-OH), 13.21 (s, 1H, C(5)-OH). ^13^C NMR (125 MHz, DMSO-d_6_) δ:15.0 (С(7)-СH_3_), 36.6 (С-6′), 61.4 (2×-OСH_3_), 72.7 (C-4′), 74.9 (C-2′), 75.3 (C-5′), 76.4 (C-3′), 97.1 (C-1′), 107.8 (C-4a), 108.5 (C-8a), 138.2 (C-6), 142.9 (C-7), 147.8 (C-3), 147.9 (C-2), 158.8 (C-8), 160.6 (C-5), 180.5 (C-1), 180.7 (C-4). HRMS (ESI): *m*/*z* [M + Na]^+^ calcd for C_19_H_22_NaO_11_S: 481.0775; found: 481.0775.

*2-(6-Deoxy-d-glucopyranos-6-ylthio)-5,8-dihydroxy-3,6,7-trimethyl-1,4-naphthoquinone* (**SAB-16**). Dark red solid; yield 78 mg (61%); *R_f_* = 0.53 (B). *α*-anomer: ^1^H NMR (500 MHz, DMSO-d_6_) δ: 2.17 (s, 6H, C(6)-CH_3_, C(7)-CH_3_), 2.36 (s, 3H, C(3)-CH_3_), 3.04 (dd, *J =* 9.9, 8.7Hz, 1H, H-4′), 3.08 (dd, *J =* 9.6, 3.6Hz, 1H, H-2′), 3.14 (dd, *J =* 14.0, 7.7 Hz, 1H, H-6′a), 3.36 (dd, *J =* 9.6, 8.7 Hz, 1H, H-3′), 3.49 (dd, *J =* 14.0, 2.6 Hz, 1H, H-6′b), 3.73 (ddd, *J =* 9.9, 7.7, 2.6 Hz, 1H, H-5′), 4.78 (d, *J =* 3.6 Hz, 1H, H-1′), 13.23 (s, 1H, C(5)-OH), 13.28 (s, 1H, C(8)-OH). ^13^C NMR (125 MHz, DMSO-d_6_) δ: 12.2 (C(6)-CH_3_, C(7)-CH_3_), 14.9 (C(3)-CH_3_), 36.5 (C-6′), 70.9 (C-5′), 72.2 (C-2′), 72.6 (C-3′), 72.9 (C-4′), 92.3 (C-1′), 108.8 (C-8a), 109.0 (C-4a), 139.9 (C-6^∗^), 140.2 (C-7^∗^), 142.8 (C-2), 145.1 (C-3), 169.0 (C-5), 169.2 (C-8), 172.3 (C-4), 172.7 (C-1). *β*-anomer: ^1^H NMR (500 MHz, DMSO-d_6_) δ: 2.17 (s, 6H, C(6)-CH_3_, C(7)-CH_3_), 2.37 (s, 3H, C(3)-CH_3_), 2.87 (dd, *J =* 8.8, 7.7Hz, 1H, H-2′), 3.04 (t, *J =* 8.8Hz, 1H, H-4′), 3.07 (t, *J =* 8.8 Hz, 1H, H-3′), 3.15 (dd, *J =* 14.0, 7.6 Hz, 1H, H-6′a), 3.25 (ddd, *J =* 8.8, 7.6, 2.5 Hz, 1H, H-5′), 3.48 (dd, *J =* 14.0, 2.5 Hz, 1H, H-6′b), 4.23 (d, *J =* 7.7 Hz, 1H, H-1′), 13.23 (s, 1H, C(5)-OH), 13.29 (s, 1H, C(8)-OH). ^13^C NMR (125 MHz, DMSO-d_6_) δ: 12.2 (C(6)-CH_3_, C(7)-CH_3_), 14.9 (C(3)-CH_3_), 36.3 (C-6′), 72.5 (C-4′), 74.8 (C-2′), 75.3 (C-5′), 76.2 (C-3′), 96.9 (C-1′), 108.9 (C-8a), 109.1 (C-4a), 140.0 (C-6*), 140.2 (C-7*), 142.4 (C-2), 145.4 (C-3), 169.2 (C-5), 169.5 (C-8), 172.1 (C-4), 172.5 (C-1). HRMS (ESI): *m*/*z* [M + Na]^+^ calcd for C_19_H_22_NaO_9_S: 449.0877; found: 449.0873. *—The assignment of signal is ambiguous.

*2-(6-Deoxy-d-glucopyranos-6-ylthio)-5,8-dihydroxy-3-ethyl-6,7-dimethyl-1,4-naphthoquinone* (**SAB-18**). Dark red solid; yield 82 mg (62%); *R_f_* = 0.56 (B). *α*-anomer: ^1^H NMR (500 MHz, DMSO-d_6_) δ: 1.09 (t, *J =* 7.5 Hz, 3H, -CH_2_CH_3_), 2.16 (s, 6H, C(6)-CH_3_, C(7)-CH_3_), 2.92 (q, *J =* 7.5 Hz, 2H, -CH_2_CH_3_), 3.03 (dd, *J =* 9.9, 8.7Hz, 1H, H-4′), 3.10 (dd, *J =* 9.6, 3.6Hz, 1H, H-2′), 3.17 (dd, *J =* 14.0, 7.7 Hz, 1H, H-6′a), 3.36 (dd, *J =* 9.6, 8.7 Hz, 1H, H-3′), 3.50 (dd, *J =* 14.0, 2.6 Hz, 1H, H-6′b), 3.75 (ddd, *J =* 9.9, 7.7, 2.6 Hz, 1H, H-5′), 4.79 (d, *J =* 3.6 Hz, 1H, H-1′), 13.24 (s, 1H, C(5)-OH), 13.31 (s, 1H, C(8)-OH). ^13^C NMR (125 MHz, DMSO-d_6_) δ: 12.2 (C(6)-CH_3_, C(7)-CH_3_), 13.3 (-CH_2_CH_3_), 21.9 (-CH_2_CH_3_), 36.5 (C-6′), 70.9 (C-5′), 72.2 (C-2′), 72.6 (C-3′), 73.0 (C-4′), 92.3 (C-1′), 108.9 (C-8a), 109.3 (C-4a), 140.2 (C-6*), 140.6 (C-7*), 142.0 (C-2), 149.9 (C-3), 170.5 (C-4*), 170.5 (C-5*), 170.7 (C-8*), 171.6 (C-1). *β*-anomer: ^1^H NMR (500 MHz, DMSO-d_6_) δ: 1.09 (t, *J =* 7.5 Hz, 3H, -CH_2_CH_3_), 2.16 (s, 6H, C(6)-CH_3_, C(7)-CH_3_), 2.87 (dd, *J =* 8.8, 7.7 Hz, 1H, H-2′), 2.92 (q, *J =* 7.5 Hz, 2H, -CH_2_CH_3_), 3.03 (t, *J =* 8.8 Hz, 1H, H-4′), 3.07 (t, *J =* 8.8 Hz, 1H, H-3′), 3.15 (dd, *J =* 14.0, 7.6 Hz, 1H, H-6′a), 3.26 (ddd, *J =* 8.8, 7.6, 2.5 Hz, 1H, H-5′), 3.52 (dd, *J =* 14.0, 2.5 Hz, 1H, H-6′b), 4.23 (d, *J =* 7.7 Hz, 1H, H-1′), 13.24 (s, 1H, C(5)-OH), 13.33 (s, 1H, C(8)-OH). ^13^C NMR (125 MHz, DMSO-d_6_) δ: 12.2 (2C, C(6)-CH_3_, C(7)-CH_3_), 13.3 (-CH_2_CH_3_), 21.8 (-CH_2_CH_3_), 36.3 (C-6′), 72.7 (C-4′), 74.8 (C-2′), 75.4 (C-5′), 76.2 (C-3′), 96.9 (C-1′), 109.0 (C-8a), 109.3 (C-4a), 140.3 (C-6*), 140.6 (C-7*), 141.5 (C-2), 150.2 (C-3), 170.3 (C-5*), 170.7 (C-4*), 170.9 (C-8*), 171.4 (C-1). HRMS (ESI): *m*/*z* [M + Na]^+^ calcd for C_20_H_24_NaO_9_S: 463.1033; found: 463.1028. *–The assignment of signal is ambiguous.

*2-(6-Deoxy-d-glucopyranos-6-ylthio)-3,6,7-trichloro-5,8-dihydroxy-1,4-naphthoquinone* (**SAB-20**). Purple solid; yield 69 mg (47%); *R_f_* = 0.66 (B). *α*-anomer: ^1^H NMR (500 MHz, DMSO-d_6_) δ: 3.10 (m,2H,H-2′,H-4′), 3.39 (t, *J =* 9.4 Hz, 1H, H-3′), 3.48 (dd, *J =* 13.4, 7.5 Hz, 1H, H-6′a), 3.69 (dd, *J =* 13.5, 2.1 Hz, 1H, H-6′b), 3.81 (m, 1H, H-5′), 4.75 (d, *J =* 3.7 Hz, 1H, H-1′), 4.78 (s, 1H, C(4′)-OH), 5.05 (br s, 2H, C(2′)-OH, C(3′)-OH), 6.33 (br s, 1H,C(1′)-OH), 12.49 (br s, 2H, C(5)-OH, C(8)-OH). ^13^C NMR (125 MHz, DMSO-d_6_) δ: 36.5 (С-6′), 71.0 (C-5′), 72.2 (C-2′), 72.7 (C-3′), 73.0 (C-4′), 96.9 (C-1′), 111.1 (C-4a), 111.6 (C-8a), 132.3 (C-7), 132.7 (C-6), 138.4 (C-3), 148.6 (C-2), 156.7 (C-8), 157.1 (C-5), 174.0 (C-1), 178.2 (C-4). *β*-anomer: ^1^H NMR (500 MHz, DMSO-d_6_) δ: 2.83 (m, 1H, H-2′), 3.10 (m, 2H,H-3′,H-4′), 3.34 (m, 1H, H-5′), 3.48 (dd, *J* = 13.4, 7.5 Hz, 1H, H-6′a), 3.71 (dd, *J* = 13.5, 2.1 Hz, 1H, H-6′b), 4.21 (d, *J* = 7.7 1H, H-1′), 4.18 (d, *J* = 5.2 Hz, 1H, C(4′)-OH), 5.05 (br s, 2H, C(2′)-OH, C(3′)-OH), 6.48 (br s, 1H,C(1′)-OH), 12.49 (br s, 2H, C(5)-OH, C(8)-OH). ^13^C NMR (125 MHz, DMSO-d_6_) δ: 36.1 (С-6′), 72.3 (C-4′), 74.7 (C-2′), 75.3 (C-5′), 76.1 (C-3′), 92.4 (C-1′), 110.9 (C-4a), 111.5 (C-8a), 132.2 (C-7), 132.6 (C-6), 138.0 (C-3), 148.6 (C-2), 156.2 (C-8), 156.9 (C-5), 173.3 (C-1), 177.5 (C-4). HRMS (ESI): *m*/*z* [M + Na]^+^ calcd for C_16_H_13_Cl_3_NaO_9_S: 508.9238; found: 508.9234.

*2-(6-Deoxy-d-glucopyranos-6-ylthio)-5,8-dihydroxy-1,4-naphthoquinone* (**SAB-22**). Purple solid; yield 75 mg (65%); *R_f_* = 0.48 (B). *α*-anomer: ^1^H NMR (500 MHz, DMSO-d_6_) δ: 3.06 (dd, *J =* 13.6, 7.6 Hz, 1H, H-6′a), 3.08 (m, 1H, H-4′), 3.18 (m, 1H, H-2′), 3.34 (dd, *J =* 13.6, 2.1Hz, 1H, H-6′b), 3.44 (m, 1H, H-3′), 3.86 (m, 1H, H-5′), 4.56 (d, *J =* 6.6Hz, 1H, C(2′)-OH), 4.77 (d, *J =* 4.9Hz, 1H, C(3′)-OH), 4.92 (t, *J =* 4.2 Hz, 1H, H-1′), 5.29 (d, *J =* 5.4Hz, 1H, C(4′)-OH), 6.42 (d, *J =* 4.7Hz, 1H, C(1′)-OH), 6.87 (s, 1H, H-3), 7.34 (d, *J =* 9.4 Hz, 1H, H-7), 7.40 (d, *J =* 9.4Hz, 1H, H-6), 11.86 (s, 1H, C(8)-OH), 12.59 (s, 1H, C(5)-OH). ^13^C NMR (125 MHz, DMSO-d_6_) δ: 32.9 (С-6′), 69.1 (C-5′), 72.2 (C-2′), 72.7 (C-3′), 73.6 (C-4′), 92.6 (C-1′), 111.2 (C-4a), 111.7 (C-8a), 127.3 (C-3), 128.6 (C-7), 130.4 (C-6), 157.0 (C-2), 157.6 (C-5), 157.5 (C-8), 183.8 (C-1), 184.2 (C-4). *β*-anomer: ^1^H NMR (500 MHz, DMSO-d_6_) δ: 2.94 (m, 1H, H-2′), 3.04 (dd, *J =* 13.5, 7.9 Hz, 1H, H-6′a), 3.11 (m, 1H, H-4′), 3.16 (m, 1H, H-3′), 3.35 (dd, *J =* 13.5, 2.1 Hz, 1H, H-6′b), 3.41 (m, 1H, H-5′), 4.34 (dd, *J =* 7.5, 6.9 Hz, 1H, H-1′), 4.91 (d, *J =* 4.8 Hz, 1H, C(2′)-OH), 4.96 (d, *J =* 4.7 Hz, 1H, C(3′)-OH), 5.33 (d, *J =* 5.0 Hz, 1H, C(4′)-OH), 6.71 (d, *J =* 6.6 Hz, 1H, C(1′)-OH), 6.87 (s, 1H, H-3), 7.34 (d, *J =* 9.4 Hz, 1H, H-7), 7.40 (d, *J =* 9.4Hz, 1H, H-6), 11.86 (s, 1H, C(8)-OH), 12.59 (s, 1H, C(5)-OH). ^13^C NMR (125 MHz, DMSO-d_6_) δ: 32.6 (С-6′), 73.1 (C-4′), 73.5 (C-5′), 74.8 (C-2′), 76.2 (C-3′), 97.1 (C-1′), 111.2 (C-4a), 111.7 (C-8a), 127.4 (C-3), 128.6 (C-7), 130.4 (C-6), 155.5 (C-2), 156.9 (C-5), 157.5 (C-8), 183.8 (C-1), 184.3 (C-4). HRMS (ESI): *m*/*z* [M + Na]^+^ calcd for C_16_H_16_NaO_9_S: 407.0407; found: 407.0402.

*2-(6-Deoxy-d-glucopyranos-6-ylthio)-8-hydroxy-1,4-naphthoquinone* (**SAB-24**). Yellow solid; yield 74 mg (64%); *R_f_* = 0.46 (B). *α*-anomer: ^1^H NMR (500 MHz, DMSO-d_6_) δ: 2.99 (dd, *J =* 13.4, 8.2 Hz, 1H, H-6′a), 3.09 (m, 1H, H-4′), 3.18 (m, 1H, H-2′), 3.31 (dd, *J =* 13.4, 2.6 Hz, 1H, H-6′b), 3.44 (m, 1H, H-3′), 3.86 (m, 1H, H-5′), 4.56 (d, *J =* 6.6 Hz, 1H, C(2′)-OH), 4.78 (d, *J =* 5.0 Hz, 1H, C(3′)-OH), 4.92 (t, *J =* 4.4 Hz, 1H, H-1′), 5.28 (d, *J =* 5.3 Hz, 1H, C(4′)-OH), 6.42 (d, *J =* 4.6 Hz, 1H, C(1′)-OH), 6.81 (s, 1H, H-3), 7.30 (d, *J =* 8.4, 0.8 Hz, 1H, H-7), 7.50 (d, *J =* 7.4, 0.8Hz, 1H, H-5), 7.75 (dd, *J =* 8.4, 7.4 Hz, 1H, H-6), 11.46 (s, 1H, C(8)-OH). ^13^C NMR (125 MHz, DMSO-d_6_) δ: 32.7 (С-6′), 69.2 (C-5′), 72.2 (C-2′), 72.7 (C-3′), 73.6 (C-4′), 92.5 (C-1′), 114.9 (C-8a), 118.7 (C-5), 123.5 (C-7), 127.7 (C-3), 132.1 (C-4a), 137.3 (C-6), 154.6 (C-2), 160.5 (C-8), 180.4 (C-4), 186.2 (C-1). *β*-anomer: ^1^H NMR (500 MHz, DMSO-d_6_) δ: 2.94 (m, 1H, H-2′), 2.98 (m, 1H, H-4′), 3.03 (dd, *J =* 13.4, 7.7 Hz, 1H, H-6′a), 3.14 (m, 1H, H-3′), 3.33 (dd, *J =* 13.4, 2.3 Hz, 1H, H-6′b), 3.40 (m, 1H, H-5′), 4.33 (dd, *J =* 7.5, 6.6 Hz, 1H, H-1′), 4.91 (d, *J =* 4.8 Hz, 1H, C(2′)-OH), 4.96 (d, *J =* 4.7 Hz, 1H, C(3′)-OH), 5.33 (d, *J =* 5.1 Hz, 1H, C(4′)-OH), 6.71 (d, *J =* 6.6 Hz, 1H, C(1′)-OH), 6.81 (s, 1H, H-3), 7.30 (d, *J =* 8.4, 0.8 Hz, 1H, H-7), 7.50 (d, *J =* 7.4, 0.8Hz, 1H, H-5), 7.75 (dd, *J =* 8.4, 7.4 Hz, 1H, H-6), 11.46 (s, 1H, C(8)-OH). ^13^C NMR (125 MHz, DMSO-d_6_) δ: 32.5 (С-6′), 73.1 ( C-4′), 73.5 (C-5′), 74.8 (C-2′), 76.2 (C-3′), 97.1 (C-1′), 114.9 (C-8a), 118.7 (C-5), 123.5 (C-7), 127.8 (C-3), 132.1 (C-4a), 137.3 (C-6), 154.3 (C-2), 160.5 (C-8), 180.4 (C-4), 186.2 (C-1). HRMS (ESI): *m*/*z* [M + Na]^+^ calcd for C_16_H_16_NaO_8_S: 391.0458; found: 391.0455.

*2-(6-Deoxy-d-glucopyranos-6-ylthio)-5-hydroxy-1,4-naphthoquinone* (**SAB-26**). Yellow solid; yield 65 mg (59%); *R_f_* = 0.46 (B). *α*-anomer:^1^H NMR (500 MHz, DMSO-d_6_) δ: 3.01 (dd, *J =* 13.4, 8.1 Hz, 1H, H-6′a), 3.08 (m, 1H, H-4′), 3.18 (m, 1H, H-2′), 3.32 (dd, *J =* 13.4, 2.4Hz, 1H, H-6′b), 3.44 (m, 1H, H-3′), 3.87 (m, 1H, H-5′), 4.56 (d, *J =* 6.7Hz, 1H, C(2′)-OH), 4.77 (d, *J =* 4.9Hz, 1H, C(3′)-OH), 4.92 (t, *J =* 4.2 Hz, 1H, H-1′), 5.28 (d, *J =* 5.4Hz, 1H, C(4′)-OH), 6.42 (d, *J =* 4.8Hz, 1H, C(1′)-OH), 6.81 (s, 1H, H-3), 7.36 (d, *J =* 8.4, 1.0 Hz, 1H, H-6), 7.56 (d, *J =* 7.5, 1.0Hz, 1H, H-8), 7.71 (dd, *J =* 8.4, 7.5Hz, 1H, H-7), 12.12 (s, 1H, C(5)-OH). ^13^C NMR (125 MHz, DMSO-d_6_) δ: 32.9 (С-6′), 69.1 (C-5′), 72.2 (C-2′), 72.7 (C-3′), 73.6 (C-4′), 92.6 (C-1′), 114.4 (C-4a), 119.3 (C-8), 124.8 (C-6), 126.8 (C-3), 131.7 (C-8a), 136.1 (C-7), 156.1 (C-2), 160.5 (C-5), 181.2 (C-1), 186.6 (C-4). *β*-anomer:^1^H NMR (500 MHz, DMSO-d_6_) δ: 2.94 (m, 1H, H-2′), 3.02 (dd, *J =* 13.4, 7.9 Hz, 1H, H-6′a), 3.10 (m, 1H, H-4′), 3.16 (m, 1H, H-3′), 3.35 (dd, *J =* 13.4, 2.3 Hz, 1H, H-6′b), 3.41 (m, 1H, H-5′), 4.34 (dd, *J =* 7.6, 6.6 Hz, 1H, H-1′), 4.91 (d, *J =* 4.6 Hz, 1H, C(2′)-OH), 4.95 (d, *J =* 4.8 Hz, 1H, C(3′)-OH), 5.33 (d, *J =* 5.1 Hz, 1H, C(4′)-OH), 6.70 (d, *J =* 6.7 Hz, 1H, C(1′)-OH), 6.81 (s, 1H, H-3), 7.36 (d, *J =* 8.4, 1.0 Hz, 1H, H-6), 7.56 (d, *J =* 7.5, 1.0Hz, 1H, H-8), 7.71 (dd, *J =* 8.4, 7.5Hz, 1H, H-7), 12.12 (s, 1H, C(5)-OH). ^13^C NMR (125 MHz, DMSO-d_6_) δ: 32.6 (С-6′), 73.1 ( C-4′), 73.5 (C-5′), 74.8 (C-2′), 76.2 (C-3′), 97.1 (C-1′), 114.4 (C-4a), 119.3 (C-8), 124.8 (C-6), 126.9 (C-3), 131.7 (C-8a), 136.1 (C-7), 155.9 (C-2), 160.5 (C-5), 181.2 (C-1), 186.6 (C-4). HRMS (ESI): *m*/*z* [M + Na]^+^ calcd for C_16_H_16_NaO_8_S: 391.0458; found: 391.0455. * The assignment of signal is ambiguous.

### 4.2. Biology

#### 4.2.1. General Biology (Reagents and Antibodies)

Annexin-V-FITC was purchased from BD Bioscience (San Jose, CA, USA). MTT (3-(4,5-dimethylthiazol-2-yl)-2,5-diphenyltetrazolium bromide) and propidium iodide (PI) from Sigma (Taufkirchen, Germany). cOmplete™ *EASY*packs protease inhibitors cocktail and PhosSTOP™ *EASY*packs – from Roche (Mannheim, Germany). Anisomycin was from NeoCorp (Weilheim, Germany). z-VAD(OMe)-fmk was from Enzo Life Sciences (Farmingdale, NY, USA). H_2_O_2_ was from Carl Roth (Karlsruhe, Germany). CCCP (2-[2-(3-chlorophenyl)hydrazinylyidene]propanedinitrile) was from Sigma (Taufkirchen, Germany). JC-1 (5,5′,6,6′-tetrachloro-1,1′,3,3′-tetraethylbenzimidazolylcarbocyanine iodide) was from AdiloGen Life Science (Epalinges, Switzerland). *N*-acetylcystein was from MedChemExpress (Monmouth Junction, NJ, USA). Primary and secondary antibodies used are listed in Table 1.

#### 4.2.2. Cell Lines and Culture Conditions

The human prostate cancer cell lines PC-3, DU145, 22Rv1, and LNCaP, as well as human prostate non-cancer cell lines RWPE-1 and PNT2 were purchased from ATCC (Manassas, VA, USA). The human prostate cancer cell line VCaP was purchased from ECACC (Salisbury, UK). HEK 293T (human embryonic kidney cells), MRC-9 (human fibroblast cells) and HUVEC (human umbilical vascular endothelial cells, passage 11) were a gift from Prof. Sonja Loges (University Medical Center Hamburg-Eppendorf, Hamburg, Germany). All the cells had a passage number ≤ 30. Authentification of all cell lines used was recently performed by commercial service (Multiplexion GmbH, Heidelberg, Germany).

Cells were cultured as monolayers at 37 °C in a humidified atmosphere with 5% (v/v) CO_2_ in the correspondent culture medium: 10% FBS/RPMI medium (RPMI medium supplemented with Glutamax^TM^-I (gibco^®^ Life technologies^TM^, Paisley, UK) containing 10% fetal bovine serum (FBS, gibco^®^ Life technologies^TM^) and 1% penicillin/streptomycin (Invitrogen)) for PNT2, LNCaP, 22Rv1, PC-3, and DU145 and cells; 10% FBS/DMEM medium (DMEM medium supplemented with Glutamax^TM^-I (gibco^®^ Life technologies^TM^) containing 10% FBS and 1% penicillin/streptomycin (gibco^®^ Life technologies^TM^)) for VCaP, MRC-9, and HEK 293 cells; Keratinocyte Serum Free Medium (K-SFM) kit (gibco^®^ Life technologies^TM^, Paisley, UK, Cat. #17005-042) supplied with BPE and hEGF for RWPE-1 cells; and Clonetics^®^ EGM^TM^-2 SingleQuots^®^ medium (Lonza, Walkersville, MD, USA) containing 10% FBS for HUVEC cells. Cells were continuously kept in culture for a maximum of 3 months, and were regularly checked for stable phenotype and mycoplasma infection.

#### 4.2.3. MTT Assay

Cell viability was determined by MTT assay. Cells were pre-incubated overnight in 96-well plates (6 × 10^3^ cells/well in 100 μL/well). Then, the medium was replaced with fresh medium containing the determined compounds (100 μL/well), and cells were incubated for the indicated time. Next, 10 μL/well of (3-(4,5-dimethylthiazol-2-yl)-2,5-diphenyltetrazolium bromide) reagent were added. After 2–4 h of incubation, the media was aspirated and the plates were dried. Fifty microliters per well of DMSO were added to each well and the cell viability was measured using Infinite F200PRO reader (TECAN, Männedorf, Switzerland). Results were calculated by The GraphPad Prism software v. 7.05 (GraphPad Prism software Inc., La Jolla, CA, USA) and are represented as IC_50_ of the compounds against control cells treated with the solvent alone.

#### 4.2.4. Quantitative Real-Time PCR (qPCR)

GLUT-1 gene expression in different cell lines was measured by qPCR. Cells were seeded in Petri dishes (1 × 10^6^ cells per ø 10 cm dish in 5 mL of media for 22Rv1) in 10% FBS/RPMI media and incubated overnight. Cells were harvested by scratching, washed, and homogenized using QIAshredder (Cat. # 79654, QIAGEN, Hilden, Germany). The total RNA was isolated using PureLink^®^ RNA Mini Kit (Cat. # 12183018A, Invitrogen, Carlsbad, CA, USA) with the on-column DNA digestion using PureLink™ DNase (Cat. # 12185-010, Invitrogen). RNA was diluted up to 30 µL and 2 µg of RNA was transcribed into cDNA using Maxima First Strand cDNA Synthesis Kit for RT-qPCR (Cat. # K1642, Thermo Scientific, Vilnius, Lithuania). qPCR was performed using 2X KAPA SYBR FAST qPCR Master Mix Optimized for Roche LightCycler 480 (Cat. # KK4609, KAPA biosystems, Worburn, MA, USA) according to the manufacturer′s recommendations. For each reaction, 20 ng of template cDNA and 2 pmol of primers were used. The expression of human GLUT-1 and GAPDH genes were analyzed using primers, purchased from by Eurofins MWG-Biotech AG (Ebersberg, Germany). Primers sequences and melting temperatures (Tm): GLUT-1: forward AAC TCT TCA GCC AGG GTC CAC, reverse CAC AGT GAA GAT GAT GAA GAC, Tm = 60 °C; GAPDH: forward TGC ACC ACC AAC TGC TTA, reverse GAG GCA GGG ATG ATG TTC, Tm = 56 °C. The PCR conditions were 30 s 95 °C, followed by 40 cycles of 15 s 95 °C, 5 s Tm, and 26 s 72 °C (measurement of fluorescence). Melting curve analysis (10 s 95 °C, 60 s 65 °C, and 1 s 97 °C) was performed directly after PCR run. Relative gene expression was calculated using the 2^−∆∆CT^ method.

#### 4.2.5. Glucose Uptake Assay

The effects of the synthesized substance on glucose uptake were evaluated using the Glucose Uptake Cell-Based Assay Kit (Cayman chemicals, Ann Arbor, MI, USA) and two different methods. (a) Plate reader-based measurement: PC-3 cells (12 × 10^3^ cells/well) were seeded in two 96-well plates, incubated overnight, and treated with the investigated drugs in 100 µL/well of glucose-free 0.1% FBS/RPMI media for 24 h. Then, the 10 µL/well of either 2-NBDG solution (2-deoxy-2-[(7-nitro-2,1,3-benzoxadiazol-4-yl)amino]-d-glucose, 5 mg/mL, for glucose uptake measurements) or vehicle solution (for cell viability measurements) in glucose-free 0.1% FBS/RPMI media were added to each well. After 6 h of incubation, the cells were carefully washed twice with PBS (200 µL/well). Then, for glucose uptake measurements, the 100 µL/well of PBS were added and the fluorescence was measured using Infinite F200PRO reader (TECAN, Männedorf, Switzerland); or, for the cell viability measurements, 100 µL/well of media containing MTS reagent were added to the cells and viability was measured as described before [54]. Glucose uptake rate was normalized to the cell viability measured by MTS assay in order to measure the uptake rate exclusively in the viable cells. (b) FACS-based readout: The experiment was performed as described before [26]. In brief, the PC-3 cells were seeded into 12-well plates and treated with the investigated compounds for 24 h in RPMI media (glucose-free, 0.1% FBS). The plates were then incubated with 2-NBDG solution, the cells were harvested by trypsination, washed, stained with PI, and analyzed using a flow cytometry.

#### 4.2.6. Analysis of Cell Cycle Progression and DNA Fragmentation 

The effects on cell cycle progression and DNA fragmentation were analyzed by flow cytometry using PI staining as described previously [55]. Cells (2 × 10^5^ cells/well) were pre-incubated overnight in 6-well plates. Then, cells were treated with the substances in 2 mL of fresh culture media/well for 48 h. Cells were harvested by trypsination, fixed in 70% EtOH, stained with PI, and analyzed by FACS Calibur (BD Bioscience, San Jose, CA, USA). The results were quantified using BD Bioscience Cell Quest Pro v.5.2.1. software (BD Bioscience, San Jose, CA, USA). The sub-G1 population was considered to reflect apoptotic cells (containing fragmented DNA).

#### 4.2.7. Analysis of Apoptosis (Annexin-V-FITC/PI Double Staining)

To examine induction of drug-induced apoptosis FACS-based analysis with Annexin-V-FITC and propidium iodide (PI) double staining was performed as previously described [56,57]. Cells were seeded in 6-well plates (2 × 10^5^ cells/well) and incubated overnight. Treatment with the substances was performed in 2 mL fresh culture media/well for 48 h. Cells were harvested by trypsination, stained with Annexin-V-FITC and PI for 15 min, and analyzed using FACS Calibur (BD Bioscience, San Jose, CA, USA). The results were quantified using BD Bioscience Cell Quest Pro v.5.2.1. software (BD Bioscience, San Jose, CA, USA).

#### 4.2.8. Western Blotting

Cells (10^6^ cells/well) were seeded in Petri dishes (ø 6 cm) and incubated overnight. The cells were treated with the investigational drugs in fresh culture media (5 mL/dish) for 48 h. Then, cells were harvested by scratching and lysed in the lysis buffer (1% NP-40, 50 mM Tris-HCl (pH 7.6), 0.88% NaCl, 0.25% sodium cholate, 1 mM Na_3_VO_4_, 0.1 mM PMSF, protease inhibitors cocktail (cOmplete Mini EDTA-free EASYpacks, Roche, Mannheim, Germany)). Proteins were extracted and diluted with lysis buffer and loading dye, heated 5 min at 99 °C and subjected to electrophoresis in gradient Mini-PROTEAN^®^ TGX Stain-Free^TM^ gels (Bio-Rad, Hercules, CA, USA). Proteins were transferred from gel to 0.2 µm pore PVDF membrane using Trans-Blot^®^ Turbo^TM^ RTA Mini PVDF Trans kit (Bio-Rad) and Trans-Blot^®^ Turbo^TM^ Blotting System (Bio-Rad) according to the manufactures protocol. The membrane was blocked (5% BSA in 0.05% Tween-20/TBS), treated with primary and secondary antibodies, and the signals were detected using the ECL chemiluminescence system (Thermo Scientific, Rockford, IL, USA). All procedures were performed according to the manufacturer′s protocols. The antibodies used are listed in Table 1.

#### 4.2.9. Proteomics

The sample preparation and proceeding, data acquisition and analysis have been described previously [26]. In brief, 22Rv1 cells (4 × 10^6^ cells/bottle in 20 mL/bottle) were seeded and incubated in T75 culture bottles overnight. Then, the cells were treated 2 µM of **SAB-13** or equal volume with vehicle (DMSO) for 48 h. Treatment was performed in triplicates. Cells were harvested by scratching, washed with ice-cold PBS (2 × 10 mL) and flash-frozen. The samples were further proceeded (for details, see [26]) and digested by trypsin. Peptides were separated on a 25 cm C18 reversed phase column (BEH C18 Column, 130Å, 1.7 µm, 75 µm × 250 mm, Waters Corporation, Milford, MS, USA) within a 60 min gradient from 2%–30% acetonitrile (equilibration buffer: 0.1% formic acid; elution buffer: 99.9% acetonitrile and 0.1% formic acid) buffer at a flow rate of 250 nL/min using a nano-UPLC system (Acquity, Waters Corporation, Milford, MS, USA) and full recovery autosampler vials, coupled via electrospray ionization (ESI) to a tandem mass spectrometer equipped with a quadrupole and an orbitrap (QExactive, Thermo Fisher Scientific, Bremen, Germany), using MS/MS mode in DDA (data dependent acquisition) and DIA (data independent acquisition). The peptide library necessary for DIA analysis was generated by DDA-LC-MS/MS of 1 randomly selected sample per biological triplicate. The peptides were identified with the search engine Sequest (integrated in the Proteome Discoverer software version 2.0, Thermo Fischer Scientific) using the human Uniprot protein database (EMBL; Hinxton Cambridge, UK, release December 2018). All samples were acquired in DIA mode and data extraction with the peptide library was performed using the Skyline software (version 4.2, MacCoss Lab Washington University, Seattle, WA, USA). Peptides with a dot-product (dotP) of 0.85 and above were considered for further analysis and the peptide areas were summed to protein areas. Statistical analysis (data normalization, Student′s t-tests, principal component analysis, hierarchical clustering) was performed using the Perseus software (version 1.5.8, Max-Planck Institute of Biochemistry, Munich, Germany). Proteins were considered to be significantly different in abundance if a 1.5 ≤ fold change ≤ 1/1.5 and *p*-value ≤ 0.05 was detected (Student′s *t*-test).

#### 4.2.10. Bioinformatical Analysis

Bioinformatical analysis of the proteomics data was performed using the Ingenuity Pathways Analysis (IPA) (release 46901286, QIAGEN Bioinformatics, Venlo, The Netherlands). The list of significantly regulated proteins discovered by global proteome analysis were proceeded by IPA software to access protein interaction networks, gene ontology, relevant biological molecules, and processes affected by the drug, as previously described [58]. The shortest hypothetical pathway networks were constructed, showing the most relevant direct and indirect interactions of the regulated proteins and the proteins predicted to be involved in the interactions. z-Score algorithm was used to identify biological functions which are expected to be suppressed (z-score < 0) or activated (z-score > 0) upon treatment in cancer cells. The *p*-values were calculated by IPA software using the Fischer′s exact test according to the software′s protocols.

#### 4.2.11. Analysis of Intracellular ROS Level

Intracellular ROS levels were accessed using the ROS-sensitive CM-H_2_DCFDA reagent (Cat. No. C6827, Molecular probes, Invitrogen, Eugene, OR, USA). The experiment was performed as previously described with slight modifications [59]. In brief, 22Rv1 cells (10^5^ cells/well) were seeded in 12-well plates and incubated overnight. The media was changed with freshly made pre-warmed staining solution (4 µM CM-H_2_DCFDA/PBS, 0.5 mL/well) and incubated in the dark (37 °C, 5% CO_2_) for 30 min. Then, the staining solution was exchanged with 1 mL/well of pre-warmed PBS containing the investigated compounds or H_2_O_2_ (positive control) at the indicated concentrations and the cells were incubated for 2 h. Next, the cells were trypsinized, resuspended in pre-warmed PBS (200 µL/sample) and immediately analyzed by FACS according to the manufacture′s protocol.

#### 4.2.12. Analysis of Mitochondrial Membrane Potential (ΔΨ_m_)

The drop down of mitochondrial membrane potential (ΔΨ_m_) was measured using staining with ΔΨ_m_-sensitive JC-1 dye (5,5′,6,6′-Tetrachloro-1,1′,3,3′-tetraethyl-imidacarbocyanine iodide) and flow cytometry technique. Cells (10^5^ cells/well) were seeded in 12-well plates and incubated overnight. Then, the media was changed to PBS (1 mL/well) containing the investigational drugs and the cells were incubated for 2 h (37 °C, 5% CO_2_, in the dark). The cells were trypsinized, pelleted, resuspended in 100 µL of 2 µM JC-1/PBS, incubated for 1 h (37 °C, 5% CO_2_, in the dark), and analyzed by flow cytometry technique.

#### 4.2.13. Cell Fractionation

The separation of cellular nuclear, mitochondrial, and cytosolic fractions was performed using the Cell Fractionation Kit ab109719 (abcam, Cambridge, MA, USA). 22Rv1 cells (4 × 10^6^ cells/bottle) were seeded in T75 culture bottles (in 20 mL/bottle) and incubated overnight. Cells were treated for 48 h and harvested by scratching. Further procedures were performed as previously described with slight modifications [59]. Nuclear fractions were homogenized using QIAshedder kit (QUIAGEN, Hilden, Germany). Cytosolic and mitochondrial fractions were concentrated using Amicon^®^ Ultra-2 Centrifugal Filter device (Cat. No. UFC203024, Merck, Darmstadt, Germany). 

#### 4.2.14. Data and Statistical Analysis

For statistical analyses of all the datasets (apart from proteomics data), the GraphPad Prism software v. 7.05 was used (GraphPad Prism software Inc., La Jolla, CA, USA). Data are presented as mean ± SEM. The unpaired Student′s t-test or one-way ANOVA followed by Dunnett′s post-hoc tests were used for comparison of two or several groups, correspondently. Dunnett′s post-hoc test was used to compare each of a number of a multiple treatment with a single control. All experiments were performed in in triplicates (*n* = 3, biological replicates) unless otherwise stated. Differences were considered to be statistically significant (*) if *p* < 0.05.

## 5. Conclusions

We were able to synthesize novel 6-S-(1,4-naphthoquinone-2-yl)-d-glucose chimera molecules, the derivatives of the sea urchin pigments spinochromes. The compounds exhibited a Warburg effect mediated selectivity to human prostate cancer cells (including highly drug resistant cell lines) and effectively inhibited the oxidative phosphorylation. Mitochondria were identified as a primary cellular target of these compounds. The mechanism of action included mitochondrial membrane permeabilization, followed by ROS upregulation and release of cytotoxic mitochondrial proteins (AIF and cytochrome C) to the cytoplasm, which lead to the consequent caspase-9 and -3 activation, PARP cleavage and apoptosis-like cell death.

## Figures and Tables

**Table 1:** List of antibodies used.

Antibodies	Clonality	Source	Cat.-No.	Dilution	Manufacturer
anti-AIF	mAb	rabbit	#5318	1:1000	Cell Signaling
anti-Akt	pAb	rabbit	#9272	1:1000	Cell Signaling
anti-Bcl-2	pAb	rabbit	#2876	1:1000	Cell Signaling
anti-cleaved Caspase-3	mAb	rabbit	#9664	1:1000	Cell Signaling
anti-cleaved Caspase-9	mAb	rabbit	#20750	1:1000	Cell Signaling
anti-cytochrome C	mAb	rabbit	#11940	1:1000	Cell Signaling
anti-ERK1/2	mAb	mouse	#9107	1:2000	Cell Signaling
anti-GLUT1	mAb	rabbit	#12939	1:1000	Cell Signaling
anti-JNK1/2	mAb	rabbit	#9258	1:1000	Cell Signaling
anti-MEK1/2	pAb	rabbit	#9122	1:1000	Cell Signaling
anti-mouse IgG-HRP		sheep	NXA931	1:10,000	GE Healthcare
anti-p21^Waf1/Cip1^	mAb	rabbit	#2947	1:1000	Cell Signaling
anti-p38	mAb	rabbit	#9212	1:1000	Cell Signaling
anti-PARP	pAb	rabbit	#9542	1:1000	Cell Signaling
anti-phospho-Akt	mAb	rabbit	#4058	1:1000	Cell Signaling
anti-phospho-ERK1/2	mAb	rabbit	#4377	1:1000	Cell Signaling
anti-phospho-JNK1/2	mAb	rabbit	#4668	1:1000	Cell Signaling
anti-phospho-MEK1/2	mAb	rabbit	#2338	1:1000	Cell Signaling
anti-phospho-p38	mAb	rabbit	#4511	1:1000	Cell Signaling
anti-rabbit IgG-HRP		goat	#7074	1:5000	Cell Signaling
anti-Survivin	pAb	rabbit	NB500-201	1:1000	Novus
anti-α-Tubulin	mAb	mouse	T5168	1:5000	Sigma-Aldrich
anti-β-Actin-HRP	pAb	goat	sc-1616	1:10,000	Santa Cruz

## Supplementary Materials

The following are available online at https://www.mdpi.com/1660-3397/18/5/251/s1, Table S1: List of proteins regulated in 22Rv1 cells under the SAB-13 treatment and discovered by proteomics.
